# Application of a Blood–Brain Barrier Organ-on-a-Chip Model for Assessment of Countermeasure Efficiency Against Eastern Equine Encephalitis Virus

**DOI:** 10.3390/v18050548

**Published:** 2026-05-09

**Authors:** Niloufar A. Boghdeh-Olson, Michael D. Barrera, Clayton M. Britt, David K. Schaffer, Jacquelyn A. Brown, John P. Wikswo, Aarthi Narayanan

**Affiliations:** 1 Biomedical Research Laboratory, Institute for Biohealth Innovation, George Mason University, Manassas, VA 20110, USA; nboghdeh@gmu.edu (N.A.B.-O.); mbarrer@gmu.edu (M.D.B.); 2 School of Systems Biology, College of Science, George Mason University, Manassas, VA 20110, USA; 3 Vanderbilt Institute for Integrative Biosystems Research and Education, Vanderbilt University, Nashville, TN 37212, USA; clayton.britt@vanderbilt.edu (C.M.B.); d.schaffer@vanderbilt.edu (D.K.S.); john.p.wikswo@vanderbilt.edu (J.P.W.); 4 Department of Physics and Astronomy, Vanderbilt University, Nashville, TN 37235, USA; 5 Organ Pathobiology and Therapeutics Institute, University of Pittsburgh, Pittsburgh, PA 15261, USA; jab890@pitt.edu; 6 Department of Biomedical Engineering, Vanderbilt University, Nashville, TN 37235, USA; 7 Department of Molecular Physiology and Biophysics, Vanderbilt University, Nashville, TN 37232, USA; 8 Department of Biology, College of Science, George Mason University, Fairfax, VA 22030, USA

**Keywords:** alphavirus, eastern equine encephalitis virus, blood–brain barrier, organ-on-a-chip, neurovascular unit, omaveloxolone, barrier disruption, inflammation

## Abstract

Infection by neurotropic alphaviruses such as the Eastern equine encephalitis virus (EEEV) causes extensive inflammation in the central nervous system and tissue damage, including disruption of the blood–brain barrier (BBB). Neuroinflammation and BBB disruption following infection are critical pathological considerations for the development of robust countermeasure strategies. Encephalitic disease resulting from EEEV infection currently lacks FDA-approved therapeutic intervention strategies, thus exposing a major capability gap in the ability to address the global health burden that could result from alphavirus infections. In this manuscript, we present a gravity-flow Neurovascular Unit (gNVU) model of the human BBB that may be used for modeling EEEV-induced neuropathology and evaluating countermeasures. The data generated using this model show that EEEV infection causes a time-dependent disruption of BBB integrity and increases the inflammatory load in a manner that correlates with an increase in the viral load. The data also show that the route of introduction of the pathogen has an impact on the pathology measured, with infection through the brain side eliciting a greater inflammatory outcome than infection through the vascular route. Overall, the included data support the utility of this organ-on-a-chip (OOC) platform of the human BBB in understanding encephalitic disease caused by neurotropic viruses and evaluation of therapeutic intervention strategies.

## 1. Introduction

The Eastern equine encephalitis virus (EEEV) is a New World (NW) alphavirus belonging to the *Togaviridae* family [1,2]. EEEV is a single-stranded, positive-sense RNA virus mainly transmitted through mosquito bites [1,2]. NW or encephalitic alphaviruses, including EEEV, Venezuelan equine encephalitis virus (VEEV), and Western equine encephalitis virus, are notable for their neurotropism and high mortality in humans and animals, thus posing a One Health subject of concern [3,4]. NW alphaviruses readily invade the central nervous system (CNS), infecting cells of the blood–brain barrier (BBB) and neurons, replicating efficiently in CNS tissues that contribute to a severe neurological disease state [3,5,6,7].

The BBB is a highly specialized and selective interface that maintains the CNS homeostasis and regulates the exchange of molecules and ions between the blood and brain cells [8,9,10]. The BBB is a central component of the neurovascular unit (NVU), a complex multicellular system composed of brain microvascular endothelial cells, pericytes, astrocytes, and extracellular matrix components [8,10,11]. The endothelial cells form the core of the barrier interconnected by tight junction proteins, such as claudins, occludins, and zonula occludens, that restrict permeability [8,9]. Pericytes envelop the endothelium, contributing to vascular stability and regulating the barrier permeability, while astrocytes cover the abluminal surface of the capillaries secreting signaling molecules that support endothelial integrity and facilitate neurovascular cell signaling [8,9]. In addition to its structural role, the BBB is an active immunological interface. The endothelial and glial cells express pattern recognition receptors (PRRs), such as toll-like receptors (TLRs) and RIG-I-like receptors (RLRs), that detect viral components and initiate innate antiviral responses [12,13]. Activation of these pathways induces the expression of cytokines and chemokines, recruiting peripheral immune cells to the CNS and modulating local inflammatory responses [12,13,14,15]. While their activation is critical for controlling viral invasion and coordinating antiviral immunity, excessive or sustained inflammatory activation can disrupt the BBB integrity, contributing to viral entry into the CNS and exacerbation of neuroinflammation and neural injury, as observed during infections with neurotropic alphaviruses [3,12,13,16].

Previously, using a novel gravity-flow neurovascular unit (gNVU), a three-dimensional organ-on-a-chip (OOC) model representing the human blood–brain barrier (BBB), we established a VEEV infection model in the context of the attenuated strain VEEV-TC83 and the virulent Trinidad Donkey strain VEEV-TrD [17]. We also demonstrated that omaveloxolone (OMA), a host directed small molecule activator of Nrf2, preserved BBB integrity and inhibited viral replication in VEEV-infected gNVUs [17]. In an independent manuscript, we published that OMA treatment exerted an inhibitory effect on EEEV infection in monocultures of the cells of the BBB [18]. In this study, we extended the scope of our investigation to establish a gNVU infection model for EEEV with two routes of infection, namely, the vascular route of infection (VRI) and brain route of infection (BRI). We utilized these infection models to assess the effectiveness of OMA as a countermeasure strategy against EEEV. Our results revealed that both models of EEEV infection led to robust viral replication in the gNVU, disruption of barrier integrity, and induction of proinflammatory cytokines. The data indicate that OMA treatment contributed to the rescue of barrier integrity in infected units and modulated EEEV and inflammatory load, albeit being of modest statistical significance in some cases, especially in the context of cytokine levels. Overall, our data support the utilitarian value of this platform to understand neuropathology resulting from neurotropic viral infections, and the evaluation of countermeasure approaches.

## 2. Materials and Methods

### 2.1. Small Molecule and Drug Treatment Studies

Omaveloxolone (OMA) was purchased from MedChemExpress (RTA408, Cat. HY-12212, Monmouth Junction, NJ, USA), solubilized in DMSO, and stored at −80 °C as master stocks. For treatment of gNVUs, stocks were used to prepare a 0.5 µM concentration of OMA in a culture medium (working solution) and added to the vascular inlet side once every 24 h.

### 2.2. Cell Types and Virus

#### 2.2.1. Cells and Cell Culture

Primary human brain microvascular endothelial cells (HBMVECs) (Cell Systems, Cat. ACBRI 376, Kirkland, WA, USA) were maintained in endothelial basal medium (EBM)-2 (Lonza, Cat. CC-3162, Basel, Switzerland) supplemented with manufacturer’s endothelial growth bullet kit, 5% heat-inactivated (HI) fetal bovine serum (FBS) (Corning, Cat. 35-001-CV, Rochester, NY, USA), and 1% penicillin/Streptomycin (P/S) (Corning, Cat. 30-003-CI, Rochester, NY, USA). Primary human brain vascular pericytes (HBVP) (ScienCell, Cat. 1200, Carlsbad, CA, USA) and human fetal astroglia cells, SVGp12, (ATCC, Cat. CRL-8621, Manassas, VA, USA), were maintained in Eagle’s Minimum Essential Medium (EMEM) (ATCC, Cat. 30-2003, Manassas, VA, USA) supplemented with 10% HI-FBS and 1% P/S. Vero African Green Monkey kidney epithelial cells (ATCC, Cat. CCL-81, Manassas, VA, USA) were grown in Dulbecco’s Modified Eagle’s Medium (DMEM) (Quality Biological, Cat. 112-013,101CS, Gaithersburg, MD, USA), supplemented with 10% HI-FBS, 1% P/S, and 1% L-glutamine (Corning, Cat. 25-005-CI, Rochester, NY, USA). All cells were cultured in T-25 or T-75 flasks, apart from Vero cells, which were cultured in T-75, T-150, or T-225 flasks and maintained in 37 °C, 5% CO_2_ culture conditions.

#### 2.2.2. Virus

Eastern equine encephalitis virus (EEEV) GA97 was provided by Dr. Jonathan Jacob (MRIGlobal). All experiments involving EEEV were carried out in the Biosafety Level 3 (BSL-3) laboratories of a regional biocontainment lab, Biomedical Research Laboratory at George Mason University, in compliance with Federal Select Agent regulatory and safety requirements.

### 2.3. Assembly and Cell Loading in the gNVU

The gravity neurovascular units (gNVUs) were assembled and prepared as previously described [17]. At least 3 days prior to cell seeding, gNVUs were vacuumed for 24 h and inlet reservoirs were loaded with sterile distilled water. Sterile water was exchanged daily by removing perfused water from exit reservoirs and adding new sterile water to inlets to check for perfusion. Units that demonstrated flow irregularities and barrier leakage were excluded from the study. At least 1 day prior to seeding, chips were treated with an extracellular matrix (ECM) of 5:4:1 mix of UltraPure distilled water (Invitrogen, Cat. 10977015, Carlsbad, CA, USA), Collagen (Sigma-Aldrich, Cat. C5533, Burlington, MA, USA), and Fibronectin (Sigma-Aldrich, Cat. F1141, Burlington, MA, USA) using a 1 mL syringe and tygon tubing.

Cells were seeded as previously described, with cellular organization of each gNVU consisting of the vascular chamber loaded with HBMVECs at a density of approximately 100,000 and the brain chamber comprising 1:1 at approximately 50,000 SVGp12 cells and 50,000 HBVP cells [17]. The organization of the gNVUs is illustrated in Figure S1. For all gNVUs, the vascular media refers to HBMVECs growth medium, EBM-2 + 5% HI-FBS + 1% P/S, while the brain side media refers to EBM-2 + 1% HI-FBS + 1% P/S.

### 2.4. Infection and Treatment

**Figure 1:**
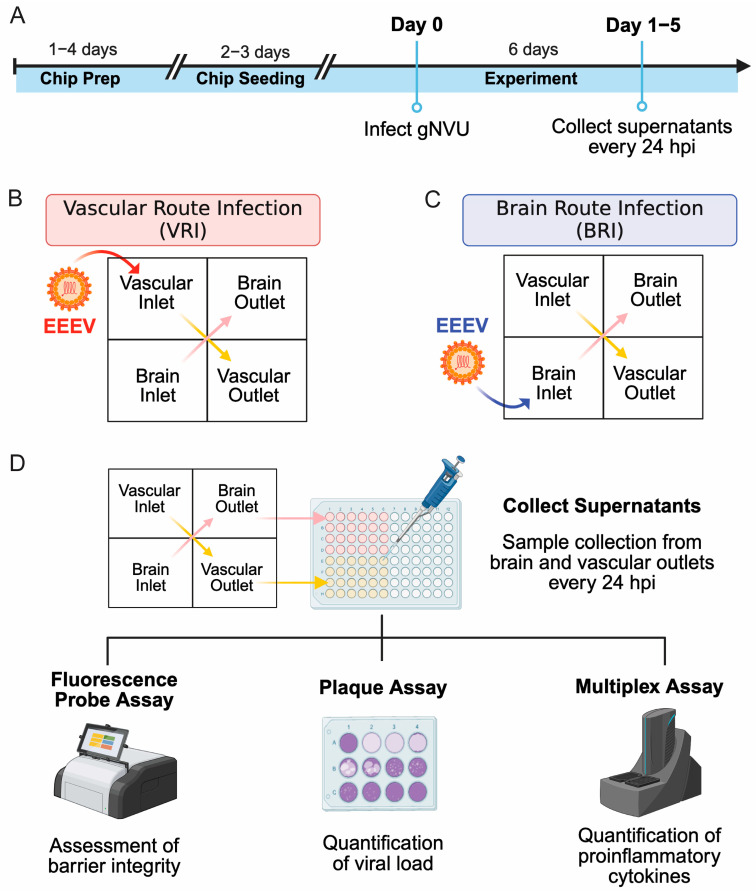
Experimental design and workflow for the establishment of EEEV gNVU infection models. (**A**) Schematic of gNVU infection experimental design. gNVUs were assembled and seeded a week prior to infection. Supernatants were collected from infected gNVUs every 24 hpi for 5 days post infection. (**B**) Illustration of the EEEV gNVU infection through the vascular route. (**C**) Illustration of the EEEV gNVU infection through the brain route. (**D**) Illustration of experimental analyses of samples collected. Supernatant samples collected from all timepoints were assessed for barrier integrity, viral load, and proinflammatory cytokines as described. Created with BioRender.com.

gNVUs were assembled and seeded a week prior to infection (Figure 1A). Units that demonstrated flow irregularities and barrier leakage were excluded from the study prior to infection. For initial establishment of infection models, EEEV at an MOI 0.1 was introduced either through a vascular inlet, referred to as the vascular route of infection (VRI), or infection was introduced through a brain inlet, referred to as the brain route of infection (BRI). Control group units refer to uninfected gNVUs. Supernatant samples were collected from infected (*n* = 3) and control (*n* = 2) gNVUs every 24 hpi for 5 days post infection. All figure data represented in color red indicate virus introduction on vascular side (VRI), while data represented in color blue indicate virus introduction on brain side (BRI) unless stated otherwise. In all figures, yellow arrows represent vascular side media or perfused supernatants collected from vascular side of chips, while pink arrows represent brain side media or perfused supernatants collected from brain side of chips, unless described otherwise.

#### 2.4.1. EEEV VRI and OMA Treatment

For the EEEV VRI gNVU model with OMA (0.5 µM) treatment, gNVUs were either infected with EEEV MOI 0.1 on the vascular inlet side and left untreated (*n* = 4), or were infected with EEEV MOI 0.1 on the vascular inlet and treated with OMA 0.5 µM on the vascular side (*n* = 4). Control group gNVUs were untreated and uninfected (*n* = 4). All figure data represented in color red indicate virus introduction on vascular side (VRI) and samples collected from infected chips, while data represented in color green indicate OMA treated chips, unless stated otherwise.

#### 2.4.2. EEEV BRI and OMA Treatment

For the EEEV BRI model, gNVU chips were infected with EEEV MOI 0.1 on the brain inlet side and left untreated (*n* = 4), or were infected on the brain inlet and treated with OMA 0.5 µM on the vascular side (*n* = 4). Control group gNVUs were again untreated and uninfected (*n* = 4). All figure data represented in color blue indicate virus introduction on brain side (BRI) and samples from infected chips, while data represented in color green indicate OMA treated chips, unless stated otherwise.

### 2.5. Barrier Permeability Assay

Barrier permeability was assessed by measuring fluorescence intensity and fluorescein isothiocyanate-dextran (FITC-dextran), and working concentrations were prepared as previously established [17]. In brief, FITC-dextran (Sigma-Aldrich, Cat. FD70S, Burlington, MA, USA) was introduced with vascular growth media on the vascular inlet. Perfused samples from brain and vascular outlets were collected every 24 h and assessed by measuring and comparing the fluorescence intensity using GloMax Microplate Reader by Promega (Promega, Cat. GM3000, Madison, WI, USA). In all figures, yellow arrows represent vascular side media or perfused supernatants collected from vascular side of chips with FITC-dextran, while pink arrows represent brain side media or perfused supernatants collected from brain side of chips, unless described otherwise. By measuring concentration in the brain compartment, the applied permeability coefficient was calculated as:

*P_app_* = *V_b_* × *C_a_*/(*C_b_* × *A* × *t*)

where *V_b_* is the brain chamber volume in cm^3^, *A* is the membrane growth area in cm^2^, *C_a_* is the initial vascular concentration of dextran in µM, *C_b_* is the brain concentration of dextran in µM and *t* is the assay time in seconds. The effective permeability of the BMEC monolayer was calculated by subtracting the permeability of an empty device according to the equation:

1/*P_total_* = 1/*P_cells_* + 1/*P_membrane_*

### 2.6. Plaque Assay

Viral load quantification was carried out by plaque assay as previously described [17]. Briefly, Vero cells were seeded in 12-well plates at 150,000 cells per well. Supernatant samples were diluted in DMEM from 10^1^ to 10^8^ and the virus-containing medium was overlaid on the cells for 1 h of infection. At 1 hpi, 1 mL of a 1:1 solution of 0.6% agarose (UltraPure Agarose, Invitrogen, Cat. 16500500, Carlsbad, CA, USA) in distilled H_2_O with 2× X eagle’s minimal essential medium (EMEM 2×) (Quality Biological, Cat. 115-073-101, Gaithersburg, MD, USA) was added to each well. The agarose was allowed to solidify at room temperature and plates were subsequently transferred to 37 °C, 5% CO_2_ culture conditions for 48 h. At 48 hpi, plates were fixed with 1 mL of 10% formaldehyde (made from 37% Formaldehyde, VWR, Cat. 10790-710, Radnor, PA, USA) overnight at room temperature. Approximately 24 h after fixation, the agar plugs were discarded and the fixed cells were stained with 1% crystal violet in 20% ethanol solution for 10–15 min. The staining solution was removed, plates were washed with distilled water, and plaques were counted for each well to determine the plaque-forming units/mL (PFU/mL) for each sample. Mean and standard deviation results are from an average of *n* = 3 or *n* = 4 samples.

### 2.7. Cytokine Quantification Assay

Cytokine levels in the perfused media on the brain and vascular compartments of the gNVU were quantified using a commercially available, multiplex cytokine assay kit as previously described [17]. In brief, supernatants were collected at the desired timepoints post infection from brain and vascular outlet comparments and stored at −80 °C until ready for the assay. The samples were assayed as duplicates with MSD V-PLEX Proinflammatory Panel Human Kit (MesoScale Discovery, Cat. K15049D-2, Rockville, MD, USA) for 10 cytokines: IFN-γ, IL-1β, IL-2, IL-4, IL-6, IL-8, IL-10, IL-12p70, IL-13 and TNF-α. The assay was performed following the manufacturer’s protocol and read using MESO QuickPlex SQ 120 (MesoScale Discovery, Rockville, MD, USA). Data analysis and quantifications were performed using the Discovery Workbench 4.0 software by MSD MesoScale Inc.

### 2.8. Statistical Analyses

All quantifications are based on data obtained from *n* = 3 or *n* = 4 samples unless indicated otherwise. Error bars in all figures indicate standard deviations. Graphs and *p*-values were designed and calculated using unpaired two-tailed *t*-tests or two-way ANOVA on GraphPad Prism 10. Significance values are indicated using asterisks for * *p* < 0.05, ** *p* < 0.01, *** *p* < 0.001, **** *p* < 0.0001, and *p* ≥ 0.05 is not significant.

## 3. Results

### 3.1. Demonstration of EEEV Infection in the gNVU

The gNVU platforms were initially assembled and prepared in Biosafety Level 2 (BSL-2) as described and moved to the BSL-3 laboratory for infection studies. The day of virus introduction was designated as day 0, and the infection was then maintained for 120 h post infection (hpi) (days 1–5) (Figure 1A). In order to investigate the dynamics of the EEEV infection across the human BBB, we established two distinct infection routes in the gNVUs; namely, a vascular route of infection (VRI) and a brain route of infection (BRI). The two EEEV infection route models were followed to mimic different infectious exposure routes. In the VRI model, EEEV was introduced through the vascular inlet of the gNVU, recapitulating a naturally acquired infection, such as in the case of mosquito bites (Figure 1B). In the BRI model, EEEV was introduced on the brain side inlet, in this case equating to an aerosol exposure route (Figure 1C). Gravity-perfused supernatants were collected every 24 h from both vascular and brain outlets of every gNVU chip to determine barrier status and viral and cytokine loads (Figure 1D).

#### 3.1.1. EEEV Infection Disrupts BBB Integrity in gNVUs

First, we assessed and compared the effect of different routes of EEEV infection on BBB integrity and viral load in the gNVU vascular and brain compartments. Prior to infection, supernatants from vascular and brain outlets of all gNVU units were assessed for baseline barrier function by quantifying FITC-dextran permeability. Only units that showed stable baseline barrier integrity (hour 0) were used for infection studies. Control uninfected units (*n* = 2) were maintained in their regular vascular and brain media. For the VRI model, vascular media containing FITC-dextran dye and virus (MOI 0.1) was introduced into the gNVU vascular inlets (*n* = 3), while brain side inlets received the respective standard medium. In contrast, for the BRI model, EEEV (MOI 0.1) was introduced from brain side inlets (*n* = 3), while the vascular side inlet received its standard medium containing FITC-dextran dye. All units were maintained at 37 °C, 5% CO_2_ conditions for 120 hpi. Once every 24 h, gravity-perfused supernatants were collected from the vascular and brain outlet compartments of all chips, while respective inlets of all chips were replenished with standard vascular and brain culture media.

**Figure 2:**
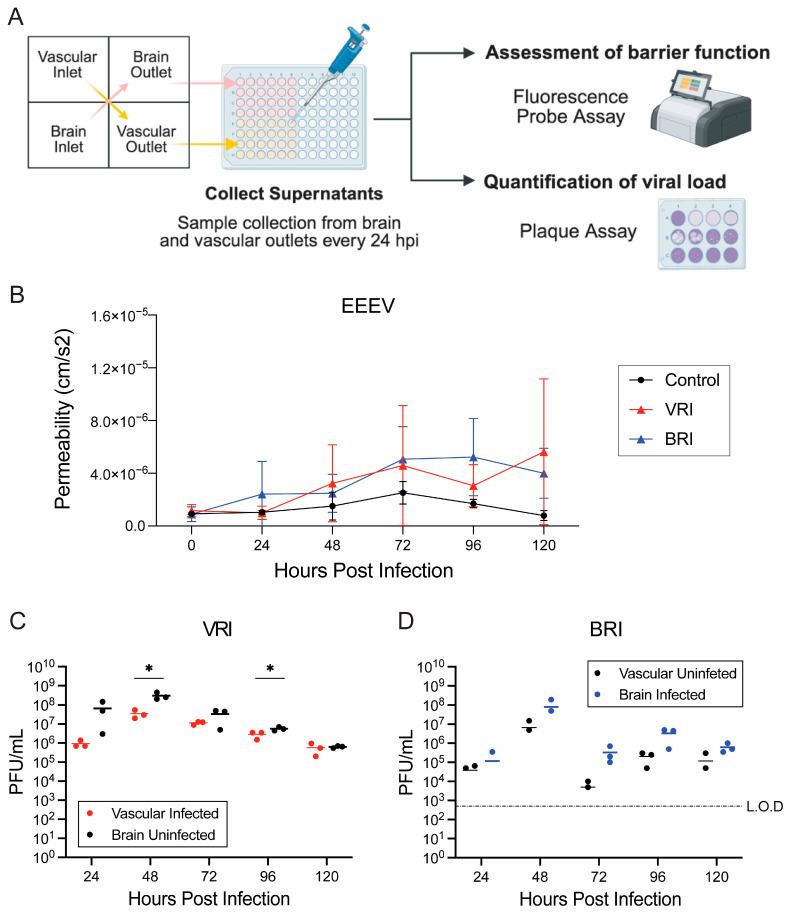
Barrier permeability and viral load quantification in the EEEV gNVU infection models. (**A**) Schematic illustration of permeability assessments and viral load quantification pipeline. (**B**) Barrier permeability was assessed by measuring fluorescence intensity of perfused FITC-dextran levels in the brain and vascular outlets. EEEV infection was introduced either through the vascular inlet (VRI) in red, or infection was introduced through the brain inlet (BRI) in blue. The control data set refers to uninfected chips. Data obtained for each infection group are averaged from *n* = 3 chips, and *n* = 2 chips for control chips. (**C**) The EEEV VRI model viral load was quantified by plaque assay using perfused supernatants collected from both vascular and brain outlet chambers at 24 h intervals up to 120 hpi. (**D**) The EEEV BRI model viral load was quantified by plaque assay using perfused supernatants collected from both vascular and brain outlet chambers at 24 h intervals up to 120 hpi. Data obtained for each infection group are averaged from *n* = 3 chips. Statistical analysis was performed using unpaired two-tailed *t*-tests on GraphPad Prism 10. Significance values are indicated using asterisks for * *p* < 0.05 and *p* ≥ 0.05 is not significant. The dashed line indicates the limit of detection (L.O.D). This figure was partially created with BioRender.com.

Barrier function and permeability of each unit were analyzed by measuring the fluorescence intensity of supernatants collected from outlet chambers during the time course (Figure 2A). The uninfected control gNVU devices maintained a stable barrier, with no detectable change in endothelial layer permeability throughout the study (Figure 2B). In contrast, EEEV infection, in both VRI and BRI models, demonstrated time-dependent barrier disruption. In the VRI model, barrier disruption was evident at 48 hpi with continued increase observed through 120 hpi (Figure 2B). Importantly, in the EEEV BRI model, permeability changes were detected as early as 24 hpi with continued high levels of barrier disruption seen at all subsequent timepoints (Figure 2B). These results demonstrate that EEEV infection disrupts the BBB integrity and increases permeability, with earlier disruption seen when infection is on the brain side. It is important to note that when looking at each individual unit in both models, the BRI model shows greater and prolonged barrier disruption compared to the VRI model, although not statistically significant (Figure S2).

The viral load in both compartments was quantified by plaque assay, measuring infectious titers from perfusates collected from vascular and brain outlets at all timepoints (Figure 2A). In the VRI model, virus was detected in both compartments at all timepoints, with the brain side samples consistently showing higher viral titers than the vascular side starting as early as 24 hpi, with statistically significant differences observed at 48 and 96 hpi (Figure 2C). Furthermore, both vascular and brain viral loads peaked at 48 hpi, the same timepoint where initial barrier disruption was observed in the VRI model (Figure 2C). Similarly, in the BRI model, the 48 hpi showed the highest viral load from both compartments, with titers from the brain side showing higher viral load across all timepoints compared to the vascular side (Figure 2D). Interestingly, compared to the VRI, not every gNVU unit with BRI had detectable infectious viral load, especially at early timepoints, although this could be attributed to subtle differences in the limit of detection (L.O.D) between chips (Figure 2D). Nevertheless, the data from both infection models show that the 48 hpi is a vital timepoint for EEEV viral replication in this gNVU platform.

#### 3.1.2. EEEV Infection Alters Cytokine Profiles in Infected gNVUs

**Figure 3:**
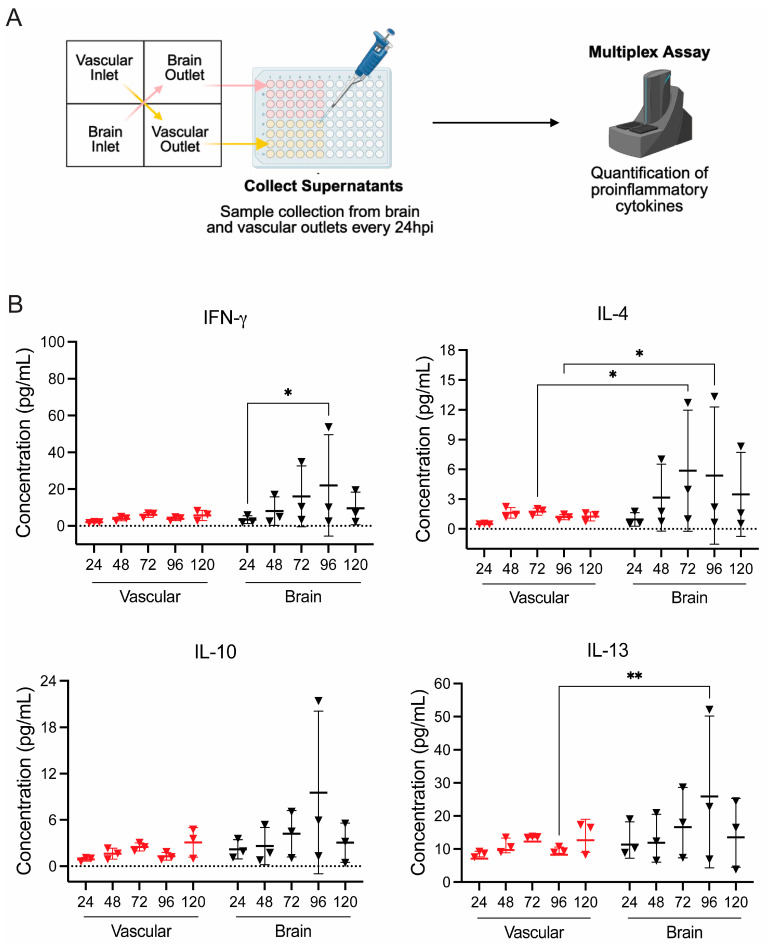
EEEV gNVU VRI inflammatory cytokines response. (**A**) Illustration of inflammatory cytokine quantifications from perfused samples collected in the VRI model. (**B**) Perfused media collected from vascular and brain outlets of VRI gNVUs were queried for 10 proinflammatory cytokines using the MSD multiplex assay. The data show four of the analyzed cytokines, namely, IFN-y, IL-4, IL-10, and IL-13. The data shown are from *n* = 3 chips, each sample collected from each timepoint analyzed as technical duplicates and averaged for each cytokine. All cytokine results were analyzed using MSD Discovery Workbench 4.0 software and represented as pg/mL concentration for each cytokine. Statistical analysis was performed using two-way ANOVA on GraphPad Prism 10. Significance values are indicated using asterisks for * *p* < 0.05 and ** *p* < 0.01, while *p* ≥ 0.05 is not significant. The complete data set of all ten inflammatory cytokines is provided as supplemental data (Figure S3). This figure was partially created with BioRender.com.

Inflammatory cytokine loads were assessed to determine whether the inflammatory responses due to EEEV infection correlated with viral replication and barrier disruption. A panel of 10 inflammatory cytokines (IFN-γ, IL-1β, IL-2, IL-4, IL-6, IL-8, IL-10, IL-12p70, IL-13 and TNF-α) was queried from vascular and brain outlets following VRI (Figure 3A). In the VRI model, EEEV infection induced a robust increase in cytokine production across all units, with higher concentrations observed on the brain side compared to the vascular side, consistent with the higher viral titers detected previously (Figure 3 and Figure S3). Remarkably, one gNVU chip showed a robust increase at 96 hpi across all ten cytokines produced on the brain side, showing a significantly higher variance in concentration compared to the other two units. Notably, both brain and vascular side IFN-γ, IL-4, IL-10 and IL-13 levels began to increase at 48 hpi, the same timepoint when barrier disruption and high viral load were seen in the VRI model (Figure 3B). Apart from one gNVU unit, the concentrations of all cytokines across both the vascular and brain side of the VRI model showed overall consistent cytokine concentrations throughout the time course starting at 48 hpi. The results demonstrate that EEEV infection in the VRI model not only causes barrier disruption and higher viral load in this model, but also intensifies inflammatory cytokine production (Figure 3 and Figure S3).

**Figure 4:**
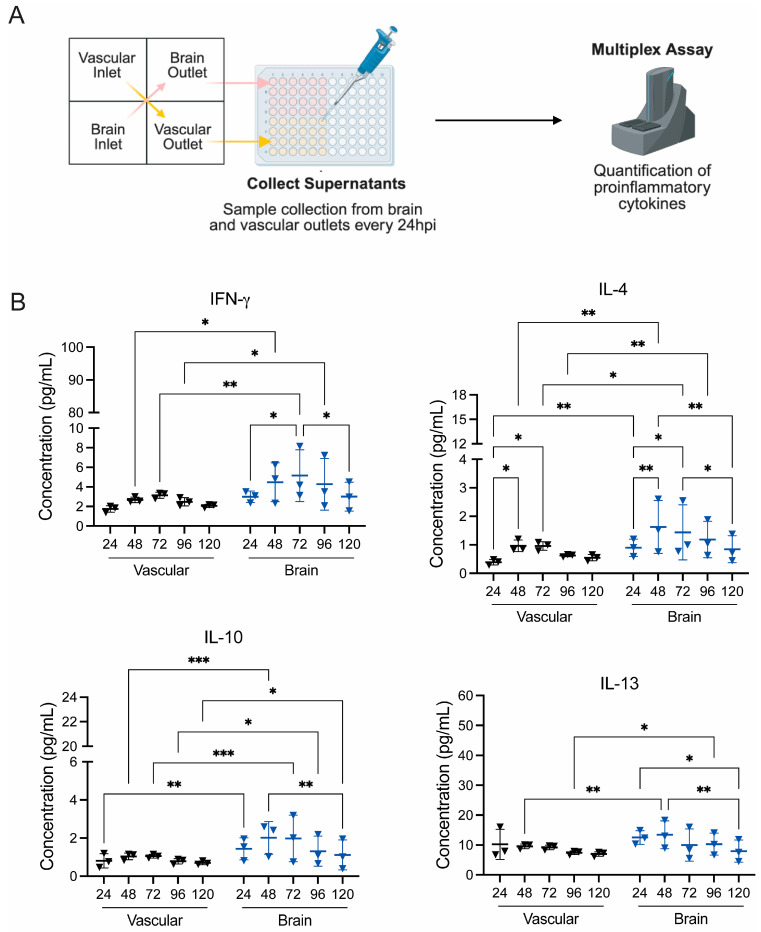
EEEV gNVU BRI inflammatory cytokines response. (**A**) Illustration of inflammatory cytokine quantifications from perfused samples collected in the BRI model. (**B**) Perfused media collected from vascular and brain outlets of BRI gNVUs were analyzed for a panel of 10 proinflammatory cytokines using the MSD multiplex assay. The data show four of the cytokines, namely, IFN-y, IL-4, IL-10, and IL-13. The data shown are from *n* = 3 chips, each sample collected from each timepoint analyzed as technical duplicates and averaged for each cytokine. All cytokine results were analyzed using MSD Discovery Workbench 4.0 software and represented as pg/mL concentration for each cytokine. Statistical analysis was performed using two-way ANOVA on GraphPad Prism 10. Significance values are indicated using asterisks for * *p* < 0.05, ** *p* < 0.01, and *** *p* < 0.001, while *p* ≥ 0.05 is not significant. The complete data set of all ten inflammatory cytokines is provided as supplemental data (Figure S3). This figure was partially created with BioRender.com.

The same cytokines were also quantified from the vascular and brain outlets following BRI (Figure 4A). In the BRI model, an overall modest cytokine response was observed. As indicated previously, we focused on four of the ten cytokines, namely, IFN-γ, IL-4, IL-10, and IL-13 (Figure 4). Although cytokine levels increased across all units starting at 48 hpi, the overall extent of response was lower than that observed in the VRI model (Figure 4 and Figure S3). Consistent with the permeability data for the BRI model, where barrier disruption was observed starting at 24 hpi, the results show that there is a statistically significant level of difference at 24 hpi for IL-4 and IL-10, with brain side concentrations being higher than the vascular side (Figure 4B). This trend was also seen across multiple cytokines for the BRI model (Figure S3). IFN-γ and IL-13 levels were higher on the brain side than the vascular side, with a statistically significant difference seen at 48 hpi (Figure 4B). One unit showed elevated IL-13 at 24 hpi in the vascular side; however, other units remained comparatively uniform and all three showed similar concentrations by 48 hpi compared to the brain side IL-13 concentrations (Figure 4B).

### 3.2. OMA Treatment Preserves BBB Integrity in EEEV VRI Model

**Figure 5:**
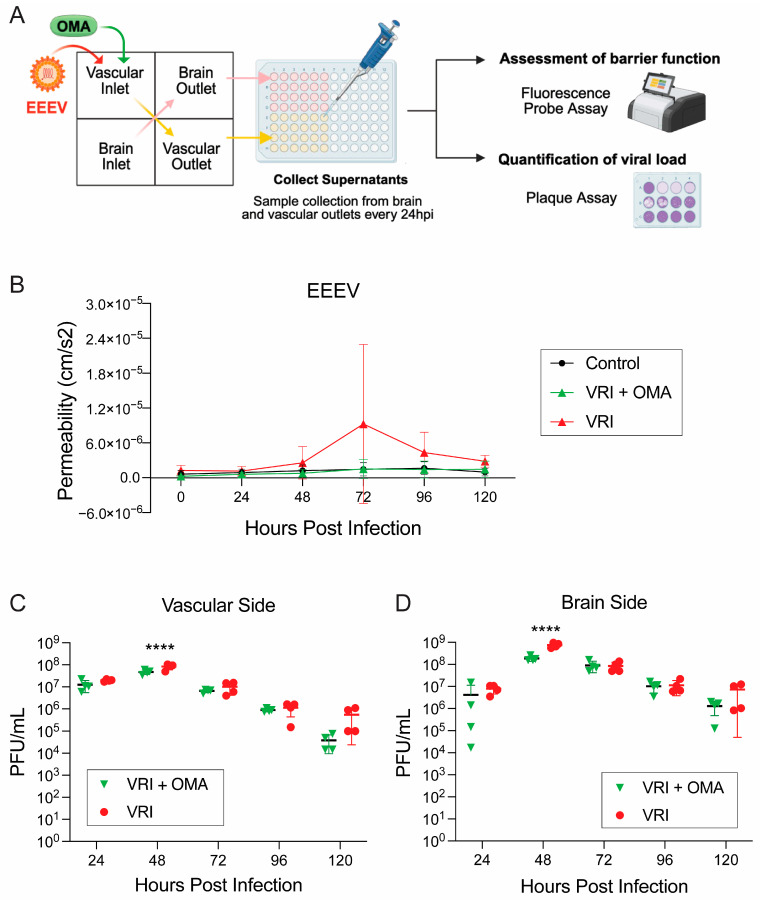
Impact of OMA treatment on barrier function and viral load in the VRI model. (**A**) Schematic of experimental design with OMA treatment in the VRI model. The gNVU chips were either infected with EEEV (MOI 0.1) on the vascular inlet side and left untreated (*n* = 4) or were infected with EEEV (MOI 0.1) on the vascular inlet and treated with OMA (0.5 µM) on the vascular side (*n* = 4). Control group gNVUs were untreated and uninfected (*n* = 4). (**B**) Barrier permeability was assessed by measuring fluorescence intensity levels of perfused media from both brain and vascular outlets at all five timepoints. The VRI untreated group is shown in red, the EEEV VRI OMA-treated group is shown in green, while the control group is seen in black. (**C**) Vascular viral load from both the untreated VRI group (red) and OMA-treated VRI group (green) was compared and quantified by plaque assay using perfused supernatants collected from vascular outlet chambers at 24 h intervals up to 120 hpi. (**D**) Brain viral load from both the untreated VRI group (red) and OMA-treated VRI group (green) was compared and quantified by plaque assay using perfused supernatants collected from brain outlet chambers at 24 h intervals up to 120 hpi. Individual timepoint comparison of viral loads is provided in Figure S4, in the Supplementary Information Section. All data represented for each group are from *n* = 4 gNVUs. Statistical analysis was performed using two-way ANOVA on GraphPad Prism 10. Significance values are indicated using asterisks for **** *p* < 0.0001, while *p* ≥ 0.05 is not significant. This figure was partially created with BioRender.com.

Next, we aimed to evaluate whether treatment of the chips with the small molecule OMA could prevent EEEV-induced barrier disruption in the VRI model following infection. Previously, we demonstrated that OMA treatment of VEEV-infected gNVUs preserved barrier integrity and inhibited viral replication [17]. Moreover, OMA was shown to robustly inhibit EEEV infection in the BBB-relevant cells in a monoculture infection model [17]. For this study, using the VRI model for EEEV, gNVU chips were either infected with EEEV (MOI 0.1) on the vascular inlet side and left untreated (*n* = 4), seen in red as the VRI group, or both EEEV and OMA (0.5 µM) were introduced on the vascular inlet side (*n* = 4), seen in green as VRI + OMA (Figure 5). Perfused supernatants were collected every 24 h over the 120 h timeframe and barrier permeability was measured using FITC-dextran (Figure 5A). The control group consisted of uninfected and untreated gNVUs. In the untreated VRI group, permeability gradually increased starting at 48 hpi, with the highest elevation observed at 72 hpi, and a gradual plateau was reached after, consistent with loss of BBB integrity seen previously in the EEEV VRI model (Figure 5B). In contrast, OMA-treated VRI units maintained stable barrier function through the entire study with permeability values comparable to uninfected control units (Figure 5B). The results support the potential of OMA to preserve barrier integrity in the EEEV VRI model.

#### 3.2.1. OMA Treatment Reduces EEEV Viral Replication in VRI Model

Following evaluation of the barrier’s integrity, we assessed the impact of OMA treatment on EEEV viral load in the VRI model. Infectious titers were quantified from perfusates collected in 24 h intervals from both the vascular and brain outlet compartments over the 120 h infection period (Figure 5A). The untreated, infected gNVUs are represented in red as VRI, while infected and OMA-treated units are represented in green as VRI + OMA, as shown in Figure 5C,D. When comparing the viral titers from the vascular perfusates, in the untreated VRI, the viral load steadily increased early on the time course, especially at 48 hpi, while OMA treatment reduced viral titers in the vascular side, with the most statistically significant decrease observed at 48 hpi (Figure 5C). Notably, viral titers in OMA-treated gNVU chips remained modestly lower than in untreated chips across all timepoints, indicating that OMA can have a reducing effect on viral replication on the vascular side (Figure 5C). Analysis of viral titers in the brain compartment revealed similar trends (Figure 5D). OMA treatment reduced viral titers in the brain compartment as well, with the statistically significant reduction observed at the 48 hpi (Figure 5D). Importantly, viral levels in OMA-treated gNVUs remained moderately lower than in untreated, infected gNVUs throughout the time course. Analysis of compartment-specific viral loads revealed statistically significant differences between vascular and brain compartments of the untreated VRI group and VRI + OMA-treated gNVUs at 24, 48, 72, and 96 hpi (Figure S4). The untreated VRI group exhibited higher viral loads in the brain side compared to the vascular side, consistent with the data presented in Figure 2C with the VRI model (Figure S4). Collectively, these results indicate that OMA treatment can reduce EEEV load in the VRI model with the brain cells continuing to exhibit higher viral titers, highlighting the critical role of brain cells in sustaining viral infection across the BBB.

#### 3.2.2. OMA Treatment of the VRI Model Modulates Inflammatory Cytokines

**Figure 6:**
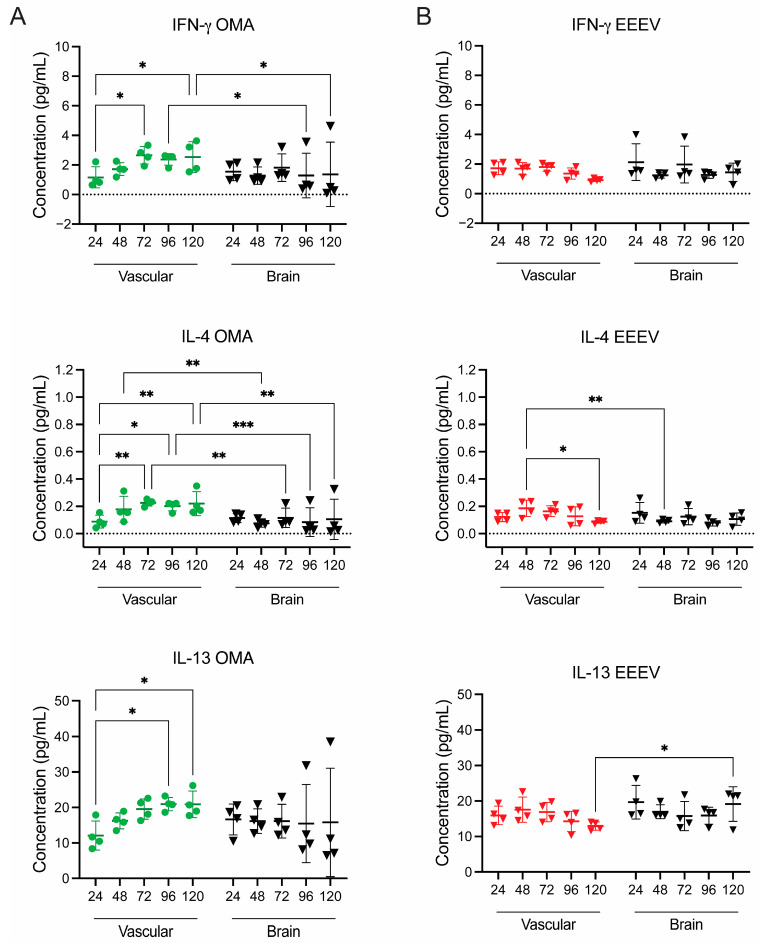
Impact of OMA treatment on cytokine responses in the EEEV VRI model. The gNVU chips were either infected with EEEV (MOI 0.1) on the vascular inlet side and left untreated (*n* = 4) or were infected with EEEV (MOI 0.1) on the vascular inlet and treated with OMA (0.5 µM) on the vascular side (*n* = 4). Perfused media collected from vascular and brain outlets of (**A**) OMA-treated VRI chips, shown in green and (**B**) untreated and VRI-infected chips, shown in red, were analyzed for a panel of 10 cytokines using MSD multiplex assay. The data show three cytokines, namely, IFN-y, IL-4, and IL-13. The data shown are from *n* = 4 chips, each sample collected from each timepoint analyzed as technical duplicates and averaged for each cytokine. All cytokine results were analyzed using MSD Discovery Workbench 4.0 software and represented as pg/mL concentration for each cytokine. Statistical analysis was performed using two-way ANOVA on GraphPad Prism 10. Significance values are indicated using asterisks for * *p* < 0.05, ** *p* < 0.01, and *** *p* < 0.001, while *p* ≥ 0.05 is not significant. The dashed line at *y* = 0 is to show standard deviation (SD) spread. The complete data set of all ten inflammatory cytokines for each group is provided as supplemental data (Figure S5).

We next aimed to assess the impact of OMA treatment on inflammatory cytokine levels in the EEEV VRI model, using the same panel of 10 inflammatory cytokines (IFN-γ, IL-1β, IL-2, IL-4, IL-6, IL-8, IL-10, IL-12p70, IL-13 and TNF-α). As previously described, gNVU chips were either infected with EEEV (MOI 0.1) on the vascular inlet side and left untreated (*n* = 4) or were infected and treated with OMA (0.5 µM) on the vascular side (*n* = 4). Perfused media collected from vascular and brain outlets for all five timepoints was quantified for ten cytokines. Figure 6 shows data for IFN-γ, IL-4, and IL-13 from vascular and brain samples of the OMA-treated group in Figure 6A and VRI untreated gNVUs in Figure 6B. The complete data set for all 10 cytokines for all units, including uninfected control units, is provided in the Supplementary Information Section (Figure S5).

In the untreated, infected VRI model, there is an initial increase noted for all cytokines starting at 24 hpi, with higher levels observed from the brain side than the vascular side samples, similar to what we observed in our earlier study for the VRI model. In the context of OMA treatment, the results show lower concentrations of IFN-γ, IL-4, and IL-13, initially at 24 hpi, compared to untreated EEEV-infected chips (Figure 6). All three cytokines IFN-γ, IL-4, and IL-13, show a statistically significant increase in concentration by 120 hpi on the vascular side with OMA treatment (Figure 6A). Furthermore, OMA treatment of the vascular side over the 5 days of infection shows a continuous increase in cytokines with concentrations starting to plateau by 120 hpi, which is the timepoint where we saw the viral titers decreasing on the vascular supernatants of OMA-treated units (Figure 6A). At the same time, we observed that OMA treatment reduced the same three cytokines on the brain side (apart from 1 chip) after 48 hpi, as was observed on the brain viral load results in the previous section (Figure 6A). Interestingly, in the untreated VRI units, the proinflammatory cytokine levels peaked at 48 hpi on the vascular side and slowly decreased over the 120 h time course (Figure 6B). Compared to the treated units, the IFN-γ, IL-4, and IL-13 concentrations from the brain side of untreated EEEV VRI units showed consistent levels across all four chips throughout the 120 h (Figure 6B).

### 3.3. OMA Treatment Preserves BBB Integrity in EEEV BRI Model

**Figure 7:**
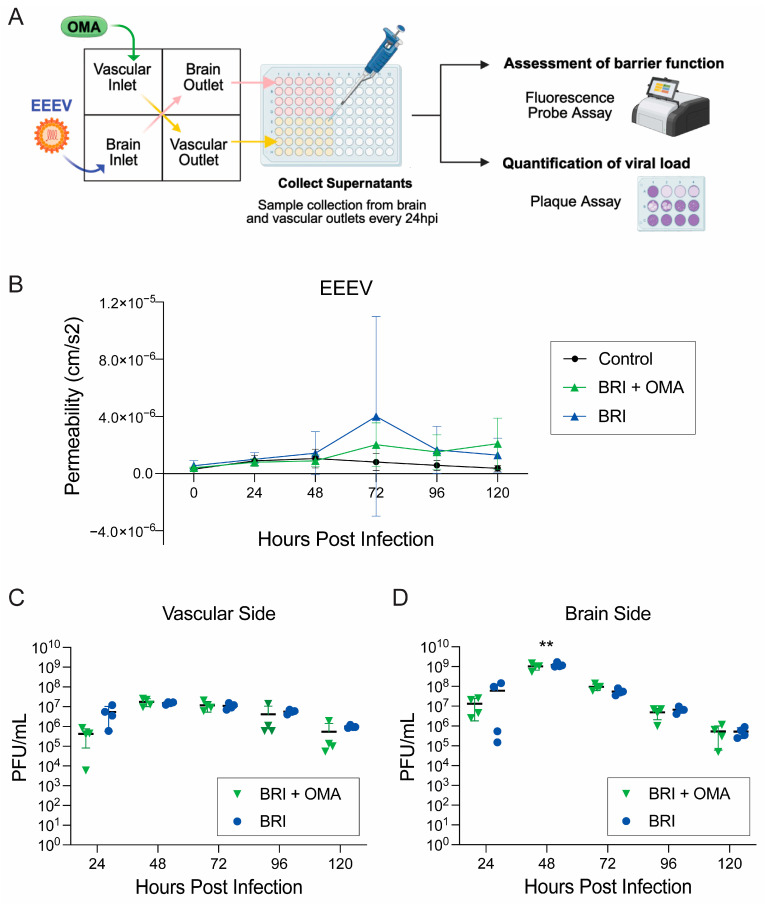
Impact of OMA treatment on barrier function and viral load in the gNVU BRI model. (**A**) Schematic of experimental design with OMA treatment in the EEEV BRI model. The gNVU chips were infected with EEEV (MOI 0.1) on the brain inlet side and left untreated (*n* = 4) or were infected with EEEV (MOI 0.1) on the brain inlet and treated with OMA (0.5 µM) on the vascular side (*n* = 4). Control group gNVUs were untreated and uninfected (*n* = 4). (**B**) Barrier permeability was assessed by measuring fluorescence intensity levels of perfused media from both brain and vascular outlets at all five timepoints. The EEEV BRI untreated group is shown in blue, the EEEV BRI OMA-treated group is shown in green, while the control group is seen in black. (**C**) Vascular viral load from untreated BRI group (blue) and OMA-treated BRI group (green) compared and quantified by plaque assay using perfused supernatants collected from vascular outlet chambers at 24 h intervals up to 120 hpi. (**D**) Brain viral load from the untreated BRI group (blue) and OMA-treated BRI group (green) was compared and quantified by plaque assay using perfused supernatants collected from brain outlet chambers at 24 h intervals up to 120 hpi. The complete data set of individual timepoint comparisons of viral loads is provided as supplemental data (Figure S6). All data represented for each group are from *n* = 4 gNVUs. Statistical analysis was performed using two-way ANOVA on GraphPad Prism 10. Significance values are indicated using asterisks for ** *p* < 0.01, while *p* ≥ 0.05 is not significant. This figure was partially created with BioRender.com.

Next, we aimed to evaluate the impact of OMA on EEEV-induced barrier disruption in the BRI model of the gNVU. Using the EEEV BRI model, gNVU chips were either infected with EEEV (MOI 0.1) on the brain inlet side and left untreated (*n* = 4), seen in blue as the BRI group, or infected on the brain side and treated with OMA (0.5 µM) on the vascular inlet side (*n* = 4), seen in green as BRI + OMA (Figure 7). Perfused supernatants were collected from outlet compartments of all units, and the inlet compartments were replenished with their designated media. All groups were replenished with brain side media on the brain inlet and vascular side media with FITC-dextran on the vascular side, apart from the treatment group vascular side inlets that included OMA (0.5 µM) in the media. Supernatants were collected at 24 h intervals over a 120 h time course and barrier permeability was measured using FITC-dextran (Figure 7A). In untreated, infected gNVUs, permeability gradually increased starting at 48 hpi, with the highest elevation observed at 72 hpi (Figure 7B). In contrast, OMA treatment in the BRI model maintained stable barrier function up to 48 hpi with permeability values comparable to uninfected control gNVUs (Figure 7B). Interestingly, unlike the results in the VRI model where OMA treatment maintained the barrier integrity throughout the 120 h of study, in the BRI model, when virus is introduced on the brain side and OMA treatment is on the vascular side, we observed a disruption to the barrier integrity starting at 72 hpi, and by 96 hpi permeability of all infected units was at a similar rate (Figure 7B). The results indicate that OMA treatment effectively preserves barrier integrity in the EEEV BRI model of gNVU early on after infection, but unlike in the VRI model, OMA is not as effective at maintaining barrier integrity after 48 hpi.

#### 3.3.1. OMA Treatment Reduces EEEV Viral Load in the BRI Model

Following the barrier assessment, the impact of OMA treatment on EEEV load was evaluated in the BRI model (Figure 7A). Viral titers were quantified from perfusates collected from the vascular and brain compartments at 24 h intervals over the 120 hpi time course. Data from the untreated, infected gNVUs are represented in blue as BRI, while infected and OMA-treated units are represented in green as BRI + OMA (Figure 7C,D). In untreated, infected gNVUs viral titers increased progressively in both compartments, similar to what was observed in the BRI model establishment studies described in Figure 2. The results from vascular perfusates show that OMA treatment reduced viral titers initially at 24 hpi, with modest reduction observed at 96 hpi and 120 hpi on the vascular side in three of the four gNVU chips, although these decreases did not show statistical significance (Figure 7C). Additionally, both untreated and treated units showed peak viral load at 48 hpi in the vascular compartment (Figure 7C). Despite the lack of statistical significance, the downward trend in viral titers aligns with the permeability data, suggesting that OMA-treatment provides partial protection during early infection and continues to modestly limit viral load at later timepoints.

Similarly, viral titers quantified from the brain compartment of both untreated and OMA-treated BRI gNVUs revealed peak viral load at 48 hpi (Figure 7D). This is again consistent with what we previously observed with both model routes of EEEV infection. Notably, OMA treatment of EEEV BRI significantly reduced viral titers in the brain compartments at 48 hpi, mirroring the results seen in the VRI model. Importantly, viral titers in OMA-treated gNVUs remained moderately lower than those in untreated EEEV-infected units across the 120 h time course, demonstrating sustained antiviral activity in the brain compartment. Nevertheless, analysis of compartment-specific viral loads between the vascular and brain side of both untreated and OMA-treated BRI gNVUS revealed statistical significance only at 48 and 72 hpi (Figure S6).

#### 3.3.2. OMA Treatment in the EEEV BRI Model Modulates Inflammatory Cytokines

**Figure 8:**
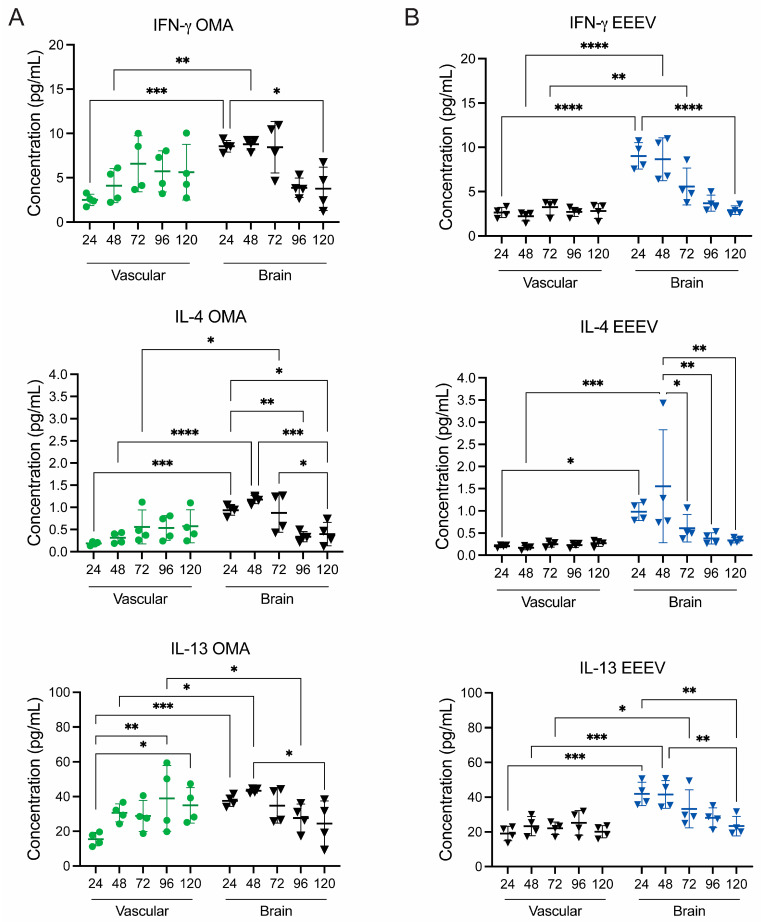
Impact of OMA treatment on inflammatory cytokine response in the EEEV BRI model. The gNVU chips were either infected with EEEV (MOI 0.1) on the brain inlet side and left untreated (*n* = 4), or were infected with EEEV (MOI 0.1) on the brain inlet and treated with OMA (0.5 µM) on the vascular side (*n* = 4). Perfused media collected from vascular and brain outlets of (**A**) OMA-treated BRI chips, shown in green and (**B**) untreated, BRI-infected chips, shown in blue, were analyzed for cytokine load using the MSD multiplex assay. The data show three cytokines, including IFN-y, IL-4, and IL-13. The data shown are from *n* = 4 chips, each sample collected from each timepoint analyzed as technical duplicates and averaged for each cytokine. All cytokine results were analyzed using MSD Discovery Workbench 4.0 software and represented as pg/mL concentration for each cytokine. Statistical analysis was performed using two-way ANOVA on GraphPad Prism 10. Significance values are indicated using asterisks for * *p* < 0.05, ** *p* < 0.01, *** *p* < 0.001, and **** *p* < 0.0001, while *p* ≥ 0.05 is not significant. The complete data set of all ten inflammatory cytokines for each group is provided as supplemental data (Figure S7).

Next, to determine whether OMA treatment also influences the inflammatory response in the EEEV BRI model, we analyzed cytokine levels from perfusates collected from the vascular and brain compartments over the 120 h infection period. As described previously, gNVU chips were either infected with EEEV (MOI 0.1) on the brain inlet side and left untreated (*n* = 4) or were infected on the brain side and treated with OMA (0.5 µM) on the vascular side inlet (*n* = 4). Perfused supernatants collected from vascular and brain outlets for all five timepoints were quantified for ten cytokines. In Figure 8, the data for IFN-γ, IL-4, and IL-13 are shown from both vascular and brain samples of the OMA-treated group in Figure 8A and BRI untreated gNVUs in Figure 8B. The complete data set for all ten inflammatory cytokines, including uninfected control units, is provided as supplemental data (Figure S7).

In OMA-treated BRI gNVUs, cytokine levels from the vascular side gradually increased over time, suggesting a mild and regulated inflammatory response during infection. For example, IFN-γ, IL-4, and IL-13 levels show an increase starting at 48 hpi in the vascular side (Figure 8A). In contrast, the brain side of OMA-treated gNVUs shows a reduction in cytokine levels over time, indicating that OMA treatment potentially mitigates excessive neuroinflammation within the BRI model (Figure S7). Notably, IL-13 cytokine levels at 24 hpi were slightly lower in both vascular and brain perfusates compared to untreated, EEEV-infected gNVUs. By 96 hpi and 120 hpi, OMA-treated units show further reduction in IL-13 levels, particularly on the brain side, inconsistent with the permeability data (Figure 8A). A similar trend was observed for IL-4, where OMA-treated gNVUs showed lower levels compared to untreated, infected units, notably at 24 hpi (Figure 8A). In untreated, EEEV-infected gNVUs, there was a pronounced increase in cytokine production on the brain side starting at 24 hpi. Comparatively, the vascular side cytokine levels remained relatively stable throughout the 120 h time course, potentially indicating a localized inflammation in the brain side (Figure 8B and Figure S7). Similar to treated samples, IL-4 and IL-13 levels peaked at 48 hpi in the brain side while the vascular levels remained consistent at all timepoints (Figure 8B). The cytokine response in the untreated infected gNVUs closely resembled the baseline BRI model cytokine profile, where brain side cytokine concentrations are typically higher than those of the vascular side.

The control group of gNVUs exhibited low cytokine levels on the vascular side at early timepoints compared to infected samples, but displayed gradual increases inconsistent with trends observed in control chips in previous data sets and may be attributed to astrocyte proliferation or metabolic overactivity over the extended culture periods within the chips (Figure S7). Overall, these findings demonstrate that OMA treatment reduced brain side proinflammatory cytokines, particularly IL-4 and IL-13, at early timepoints post infection, thereby alleviating EEEV-induced neuroinflammation in the BRI model.

## 4. Discussion

New World alphaviruses such as VEEV and EEEV can access the CNS through different routes of exposure, including from mosquito bites and aerosol exposure [7,19,20]. Following infection via a mosquito bite, the virus establishes a systemic infection [5,21]. From the peripheral sites, the virus can invade other organs, including the CNS, through the bloodstream, by directly infecting the brain microvascular endothelial cells or trafficking across the BBB through infected leukocytes [3,7,22]. In the context of an aerosol exposure, VEEV and EEEV can directly infect brain cells through a distinct pathway, namely, infection through olfactory neurons [5,7]. Once in the CNS, VEEV and EEEV replicate efficiently in neurons, which are highly permissive to infection [3,5,7]. Viral replication activates innate immune responses, including microglial activation and production of interferons and proinflammatory cytokines such as tumor necrosis factor-alpha (TNF-α), interleukin-6 (IL-6), and interferon-gamma (IFN-γ) [3,23,24]. These responses, while critical for controlling viral replication, contribute to neuroinflammation, oxidative stress, and neural apoptosis. This process, combined with rapid viral replication in the BBB cells, compromises the integrity of the NVU and promotes encephalitic pathology [3,5,23,24,25]. Importantly, studies indicate that BBB structural and functional disruption is a consequence of rather than a cause of CNS infection in the context of VEEV [7,26,27]. For example, in the intranasal infected mouse models of VEEV, viral spread to the olfactory bulb and cortex preceded detectable increase in BBB permeability, indicating that structural barrier disruption occurs later as a consequence of CNS infection [26]. Additionally, proinflammatory cytokine expression was upregulated following VEEV infection, supporting the role of inflammation in barrier breakdown [26]. Disease outcomes reflect both direct, virus-induced cytopathology and the additive damage of host immune responses, which cumulatively produce the high fatality and neurological sequelae associated with NW alphavirus encephalitis [26,27].

The results from this study provide novel insights into NW alphavirus and EEEV infection dynamics in a human BBB-relevant gNVU model and extend our prior work with VEEV-TC83 and VEEV-TrD [17]. While previous VEEV gNVU studies demonstrated that both attenuated and virulent strains could infect the BBB, disrupt the barrier integrity, and induce inflammatory cytokine production, those models only focused on the vascular route of exposure and did not assess the implications of direct brain side exposure or route-dependent differences in alphavirus infection [17]. The establishment of EEEV VRI and BRI gNVU models in this manuscript addresses this gap by simulating distinct exposure routes, allowing a comparison of how vascular versus the brain side infections may influence barrier permeability, viral load, and inflammatory responses.

In the EEEV VRI model, the results show that virus introduced through the vascular inlet induced barrier disruption beginning at 48 hpi, accompanied by peak viral titers at 48 hpi and robust proinflammatory cytokine production primarily on the brain side. These findings mirror observations from the previous work with VEEV, where vascular infection resulted in brain compartment amplification of the virus and associated cytokine response [17]. Interestingly, the EEEV VRI model elicited a more pronounced and rapid barrier disruption phenotype when compared to the VEEV models, suggesting that EEEV may display enhanced capacity to breach the BBB under conditions relevant to human infections. The elevated cytokine responses on the brain side also suggest contributions of astrocytes and pericytes in modulating neuroinflammation following infection, consistent with prior reports that these cells amplify immune signaling in response to neurotropic alphaviruses [7,15,27,28,29].

The assessment of barrier integrity as presented in this manuscript does not include conventional Transepithelial/Transendothelial Electrical Resistance (TEER) measurements. The gNVU unit was designed to be able to perform experiments with pathogens that require higher levels of biological containment (biosafety level 3). Keeping the regulatory requirements of such high containment studies in mind, the gravity-driven perfused BBB model is constructed as a fully contained system, which renders it incompatible with commercially available TEER measurement devices in its current configuration. Under this restriction, there is no direct way to pass current across the membrane rather than along the perfusion path, which prevents accurate TEER acquisition. However, the presence of continuous flow provides a distinct advantage in that we can quantify barrier function by measuring the permeability of small molecules across the BBB interface. Ongoing efforts are focused on combining imaging methods with the dextran measurements while keeping biocontainment as a central priority.

The EEEV BRI model, in which the virus was introduced directly on the brain side, exhibited earlier barrier disruption with viral titers peaking at the same 48 hpi and comparatively modest cytokine responses (Figure 4). This contrasts with what was observed with the VRI model, where vascular infection led to sustained and higher cytokine production. The limited cytokine induction in the BRI model may reflect localized inflammation without significant engagement of the vascular endothelium. These data suggest that the route of infection plays a critical role in shaping barrier integrity and inflammatory profiles, and that direct exposure to brain cells may allow the virus to circumvent vascular immune surveillance, an observation that aligns with previous reports on route-dependent neuropathogenesis of neurotropic alphaviruses [7,21,26].

Certain proinflammatory cytokines are established as critical mediators of inflammation-induced responses in the context of neurotropic viral infections, particularly at the neurovascular interface, where the endothelial activation and barrier dysfunction shape disease progression [30,31,32]. One such mediator is interleukin-8 (IL-8), a key chemokine involved in endothelial activation, neutrophil recruitment, and BBB dysfunction [31,32]. In our study, the IL-8 results showed different trends between the two routes of infection. In the BRI model, IL-8 levels gradually decreased over time in both vascular and brain compartments, suggesting direct EEEV exposure may suppress or dysregulate local IL-8 production by brain cells comprising astrocytes and pericytes (Figure S3). Astrocytes or astroglia cells are key CNS sources of IL-8, and these results highlight how glial signaling may influence the neurovascular cytokine and chemokine landscape [32,33,34]. In contrast, the VRI model led to a significant and sustained elevation of IL-8, particularly at 48 hpi, that continued up to 120 hpi, indicative of prolonged endothelial activation and vascular inflammatory signaling [32,35,36]. In the uninfected control units, IL-8 levels were elevated on the brain side and gradually increased over time, likely reflecting cytokine release associated with overgrowth and metabolic activity of astrocytes (Figure S3). This increase was also mirrored by a gradual rise in vascular side IL-8, potentially due to overgrowth signaling across the barrier from astrocytes, supporting the idea that prolonged culture or cellular stress may underlie baseline IL-8 increases in controls (Figure S3) [33,34]. Collectively, these data indicate that IL-8 responses at the BBB are strongly influenced by route of EEEV infection, with VRI eliciting a persistent endothelial-driven inflammation, whereas the BRI leads to comparatively suppressed and transient chemokine response. The different pattern of IL-8 induction across infection routes highlights how distinct cellular sources and mediators could contribute to inflammatory outcomes in a route-specific manner.

In addition to IL-8, our results included a panel of classical proinflammatory cytokines, including IL-1β, IL-6, TNF-α, and IL-12p70 (Figure S3). These cytokines are widely reported mediators of neuroinflammation and BBB dysfunction during neurotropic viral infection [30,32,37]. In our study, several of these classical inflammatory mediators show route and compartment-dependent temporal dynamics across the gNVU system. In contrast, IFN-γ, IL-4, IL-10, and IL-13 were emphasized in our analysis as representative antiviral and immunoregulatory cytokines that could capture additional aspects of host immune signaling within the gNVU BBB model. IFN-γ is a key antiviral mediator during neurotropic viral infections, while IL-10 plays an important role in limiting excessive inflammation and protecting CNS tissue. IL-4 and IL-13 are associated with immunoregulatory signaling and alternative microglial activation pathways within the CNS, highlighting potential tissue-protective mechanisms that may be relevant in future BBB models incorporating microglia.

Data variability between chips is a major challenge in OOC research. In our study, variability was observed in cytokine levels between individual gNVU units across experiments. Such variability is expected in multicellular microphysiological systems, where endothelial cells, astrocytes, and pericytes may contribute differently to cytokine production depending on local infection dynamics and cellular responses. OOC platforms can also exhibit chip to chip differences related to cell maturation, microenvironmental conditions, and viral replication kinetics. Importantly, despite this variability, consistent temporal trends were observed across replicate gNVUs, suggesting that inflammatory responses associated with the two infection routes can be captured within the gNVU chips. In order to evaluate further reproducibility of these observations, in our subsequent experiments with treatment we increased the number of biological replicates for each condition (*n* = 4). We also emphasize that the inflammatory loads reported are not indicative of specific cellular origins. Additional studies are required to specifically identify which cell type in the vascular and brain compartments are important contributors to the overall inflammatory burden.

The evaluation of OMA as a potential countermeasure for the treatment of EEEV infection further supports the relevance of this host-targeted intervention in maintaining BBB integrity and limiting viral replication. In the VRI model, OMA preserved barrier function throughout the 120 h study period and had a statistically significant impact on viral titers in both vascular and brain compartments at 48 hpi (Figure 5 and Figure S4). These results are consistent with results from the prior work with VEEV, where OMA treatment protected the BBB integrity and limited viral replication [17]. In the BRI model, OMA treatment maintained barrier integrity during early infection up to 48 hpi and reduced viral titers on the brain side, though barrier protection was less durable compared to what was observed in the VRI model (Figure 7 and Figure S6). This difference likely reflects the limited access of vascular side treatment to directly exposed brain cells in the BRI model, emphasizing the influence of infection and drug introduction route on therapeutic efficacy. Across both VRI and BRI models, OMA treatment also appeared to modulate inflammatory cytokine response in a route-dependent manner. In the VRI model, OMA initially reduced early inflammatory cytokine levels, including IFN-γ, IL-4, and IL-13, at 24 hpi compared to untreated infected chips, followed by a gradual increase in vascular side cytokines that plateaued by 120 hpi, coinciding with the reductions in viral titers. On the brain side, OMA treatment generally reduced these cytokines after 48 hpi, suggesting possible suppression of excessive neuroinflammatory signaling within the brain compartment. In contrast, in the BRI model OMA treatment was associated with gradual increases in the vascular side cytokines starting around 48 hpi while brain side cytokine levels remained the same or lower than those observed in untreated infected units. Together, these findings suggest that OMA may balance antiviral immune signaling with regulation of excessive inflammation depending on the route of infection and compartmental exposure.

Importantly, the results with OMA treatment modulating early inflammatory cytokine response are consistent with what was previously observed in VEEV models [17,18]. Moreover, Nrf2 activation, which is a known outcome of OMA treatment in different contexts, has been previously reported to have a dual role in limiting oxidative stress and regulating cytokine signaling, consistent with the results observed with OMA treatment [17,38]. Interestingly, some cytokine patterns observed in the gNVU model differ from those commonly reported in animal models of neurotropic alphavirus infection, where strong induction of proinflammatory cytokines such as IFN-γ, IL-1β, IL-6, and TNF-α is often observed in the CNS following infection with viruses such as EEEV and VEEV [23,26,30]. These differences may reflect the ability of the gNVU system to isolate neurovascular interactions between endothelial cells, astrocytes, and pericytes in the absence of infiltrating immune cells and microglia which are major contributors to cytokine amplification in vivo. Furthermore, the compartment-specific cytokine measurements enabled by the gNVU platform reveal vascular vs. brain side inflammatory dynamics that are difficult to resolve in whole animal studies, where cytokine measurements are typically performed using bulk brain tissue.

Collectively, these findings demonstrate the utility of this gNVU platform for modeling route-dependent viral neuropathogenesis and host responses, expanding on previous work with VEEV studies. Furthermore, the results of this study highlight OMA as a promising therapeutic candidate against neurotropic alphaviruses. Comparisons with the previous VEEV gNVU studies provide additional context, illustrating both conserved and virus-specific features of BBB disruption, viral replication kinetics, and inflammatory responses. The differential outcomes between VRI and BRI models emphasize the importance of infection route in therapeutic evaluation and underscore the need for complex physiologically relevant in vitro systems for preclinical therapeutic testing. Importantly, the compartmentalized architecture of the gNVU provides mechanistic insight into neurovascular interaction that cannot be readily resolved in traditional cell culture or in vivo models. While additional validation with in vivo or clinical datasets will be important for establishing full preclinical utility, the gNVU platform provides a physiologically relevant human BBB model that enables controlled investigation of neurovascular infection dynamics and therapeutic responses. Together, this work advances the development of human-relevant platforms for investigating encephalitic alphavirus infections and supports the continued integration of MPS into antiviral therapeutic discovery pipelines.

## Data Availability

Data will be made available upon request, which can be made to the corresponding author. Data will be made available after review of the request and feedback will be provided in a timely manner.

## Supplementary Materials

The following supporting information can be downloaded at: https://www.mdpi.com/article/10.3390/v18050548/s1, Figure S1: Schematic representation of the gNVU organization; Figure S2: Permeability data of individual gNVU chips of VRI and BRI models. Barrier permeability was assessed by measuring fluorescence intensity of perfused FITC-dextran levels in the brain and vascular outlets; Figure S3: EEEV gNVU VRI and BRI inflammatory cytokine expression. Perfused media collected from vascular and brain outlets gNVUs were analyzed for 10 human inflammatory cytokines (IFN-γ, IL-1β, IL-2, IL-4, IL-6, IL-8, IL-10, IL-12p70, IL-13 and TNF-α) using the MSD multiplex assay; Figure S4: Impact of OMA-treatment on viral load in the gNVU VRI model at five different timepoints; Figure S5: Impact of OMA-treatment on inflammatory cytokine response in EEEV VRI model of gNVU; Figure S6: Impact of OMA treatment on the viral load in the gNVU BRI model at five different timepoints; Figure S7: Impact of OMA treatment on the inflammatory cytokine response in the EEEV BRI model of gNVU.

## Abbreviations

The following abbreviations are used in this manuscript:

BBB	Blood–brain barrier
BRI	Brain route infection
CNS	Central nervous system
EEEV	Eastern equine encephalitis virus
FITC	Fluorescein isothiocyanate
gNVU	Gravity Neurovascular Unit
h	Hour(s)
hpi	Hours post infection
HBMVECs	Human brain microvascular endothelial cells
IL	Interleukin
Keap1	Kelch-like ECH-associated protein 1
MOI	Multiplicity of infection
NVU	Neurovascular Unit
NW	New World (alphavirus)
Nrf2	Nuclear factor erythroid-derived 2-related factor 2
OMA	Omaveloxolone
OOC	Organ-on-a-chip
PFU	Plaque forming unit
RLU	Relative light unit
VEEV	Venezuelan equine encephalitis virus
Vero	African Green monkey kidney cells
TrD	Trinidad
VRI	Vascular route infection
