# Supplementary material for: Application of a Blood–Brain Barrier Organ-on-a-Chip Model for Assessment of Countermeasure Efficiency Against Eastern Equine Encephalitis Virus
Source: Viruses. 2026 May 9;18(5):548. doi: 10.3390/v18050548 (PMC13211456; doi:10.3390/v18050548)
Supplement: Supplementary file 1 [file viruses-18-00548-s001.zip › viruses-4199962-supplementary/viruses-4199962-supplementary.pdf]

viruses

MDPI

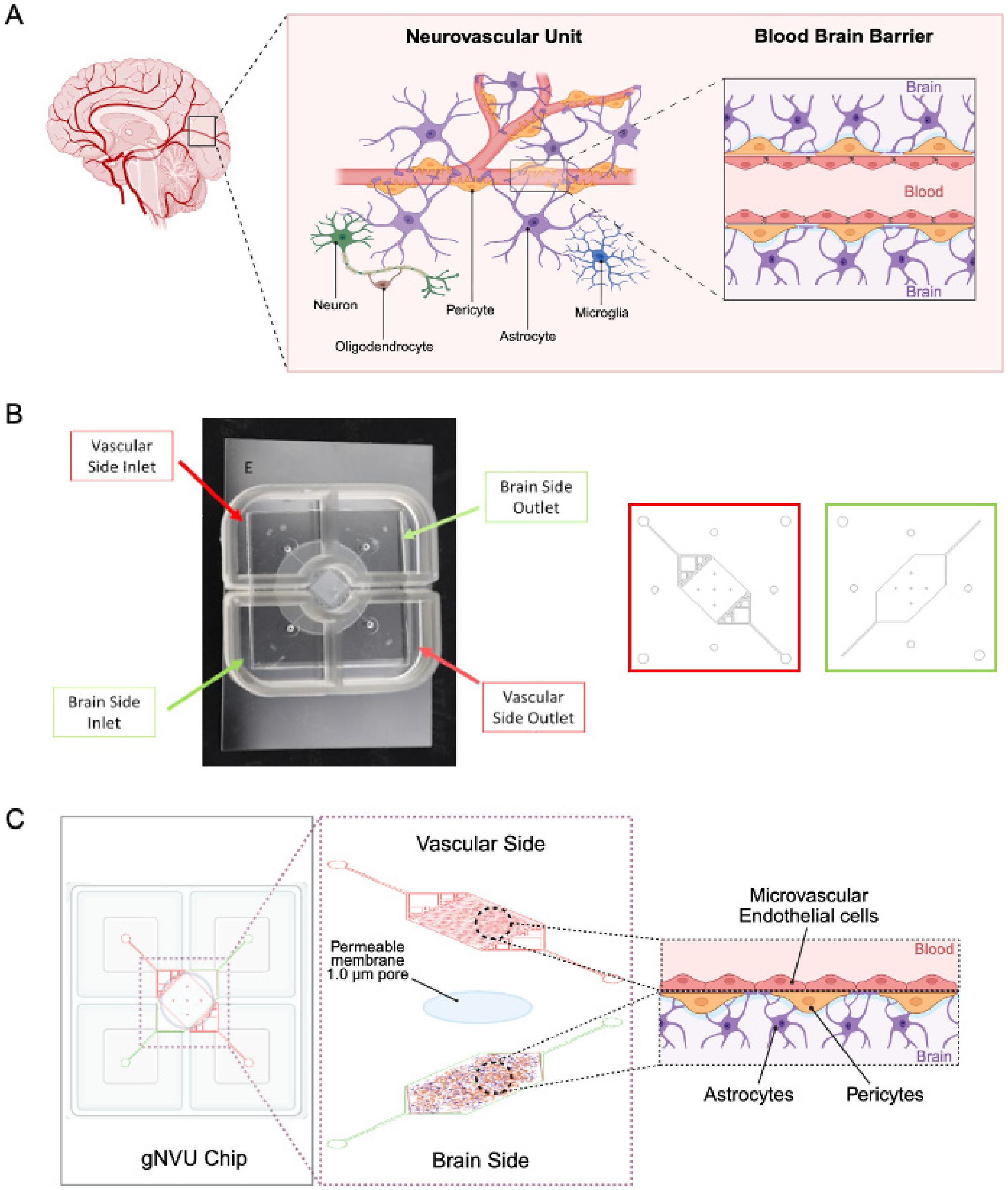

Figure S1. Schematic representation of the gNVU organization. (**A**) Illustration of a human NVU and BBB cellular organization. (**B**) Photograph image of the gNVU platform with a top-down view of vascular and brain compartment reservoirs and an illustration of each chamber. (**C**) Schematic illustration of BBB-like cellular organization of gNVU vascular chamber (brain microvascular endothelial cells) and brain chamber (astrocytes and pericytes). Figure created with BioRender.com.

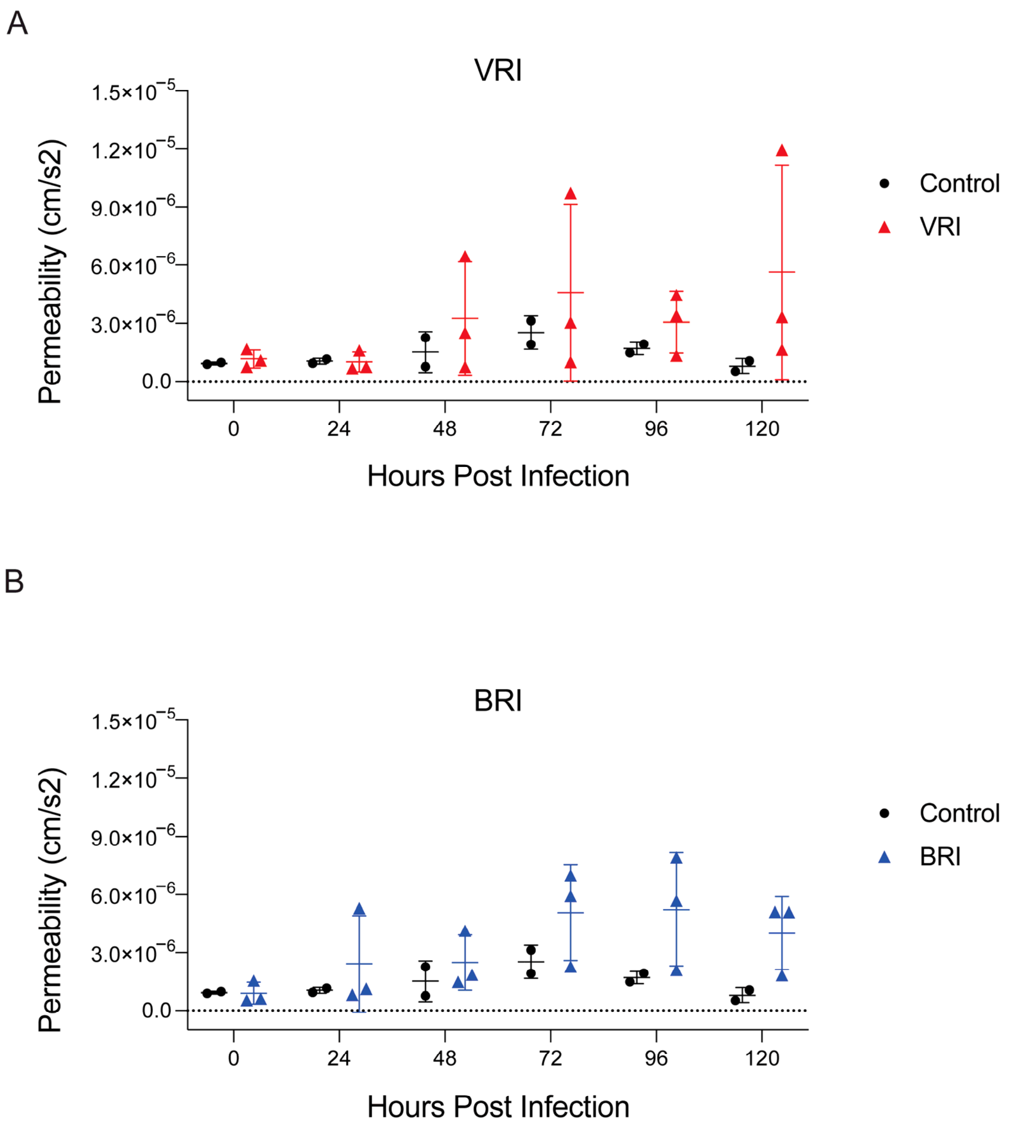

Figure S2. Permeability data of individual gNVU chips of VRI and BRI models. Barrier permeability was assessed by measuring fluorescence intensity of perfused FITC-dextran levels in the brain and vascular outlets. EEEV infection was introduced through the vascular inlet referred to as (**A**) the vascular route of infection (VRI) in red, while when infection was introduced through the brain inlet referred to as (**B**) the brain route of infection (BRI) in blue. Control data refer to gNVU uninfected chips in black in both figures (**A-B**). Data obtained for each infection group were averaged from n = 3 chips, and n = 2 chips for control chips. The dashed line at $y = 0$ is to show standard deviation (SD) spread.

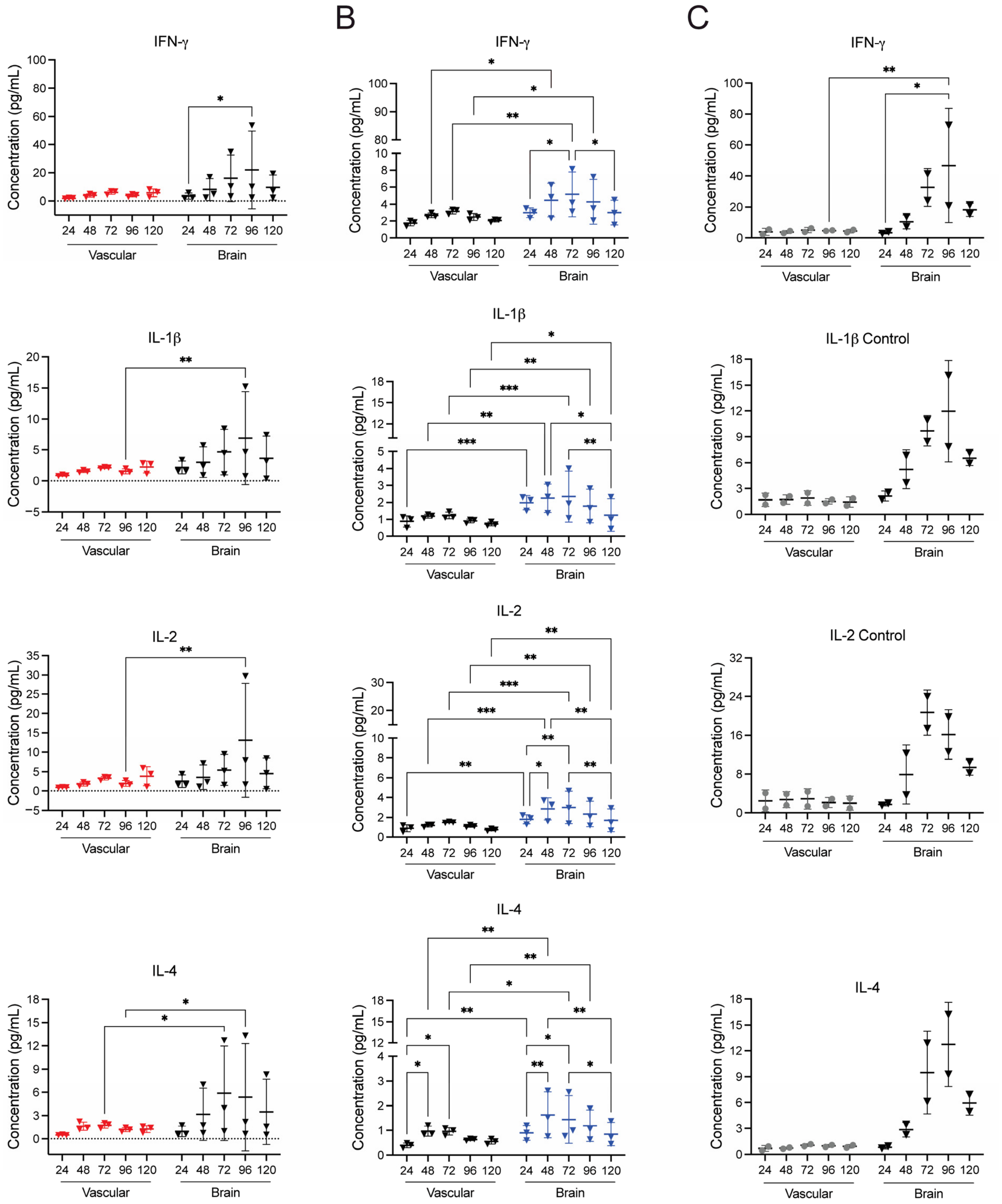
B
C
IFN-γ
IL-1β
IL-1β Control
IL-2
IL-2 Control
IL-4
Concentration (pg/mL)
24 48 72 96 120 24 48 72 96 120
Vascular
Brain

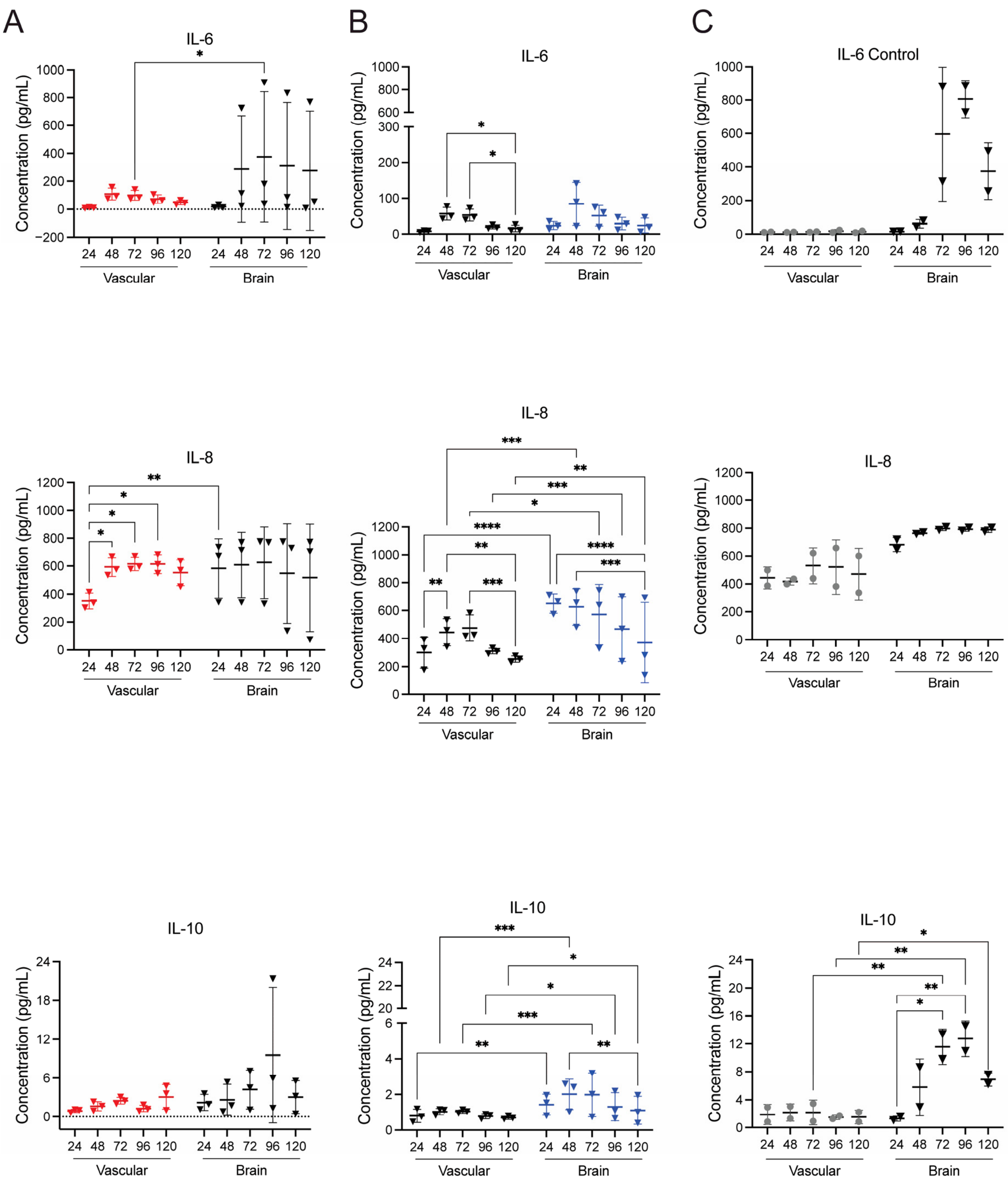
A
B
C
IL-6
IL-6
IL-6 Control
IL-8
IL-8
IL-8
IL-10
IL-10
IL-10
Concentration (pg/mL)
24 48 72 96 120 24 48 72 96 120
Vascular
Brain

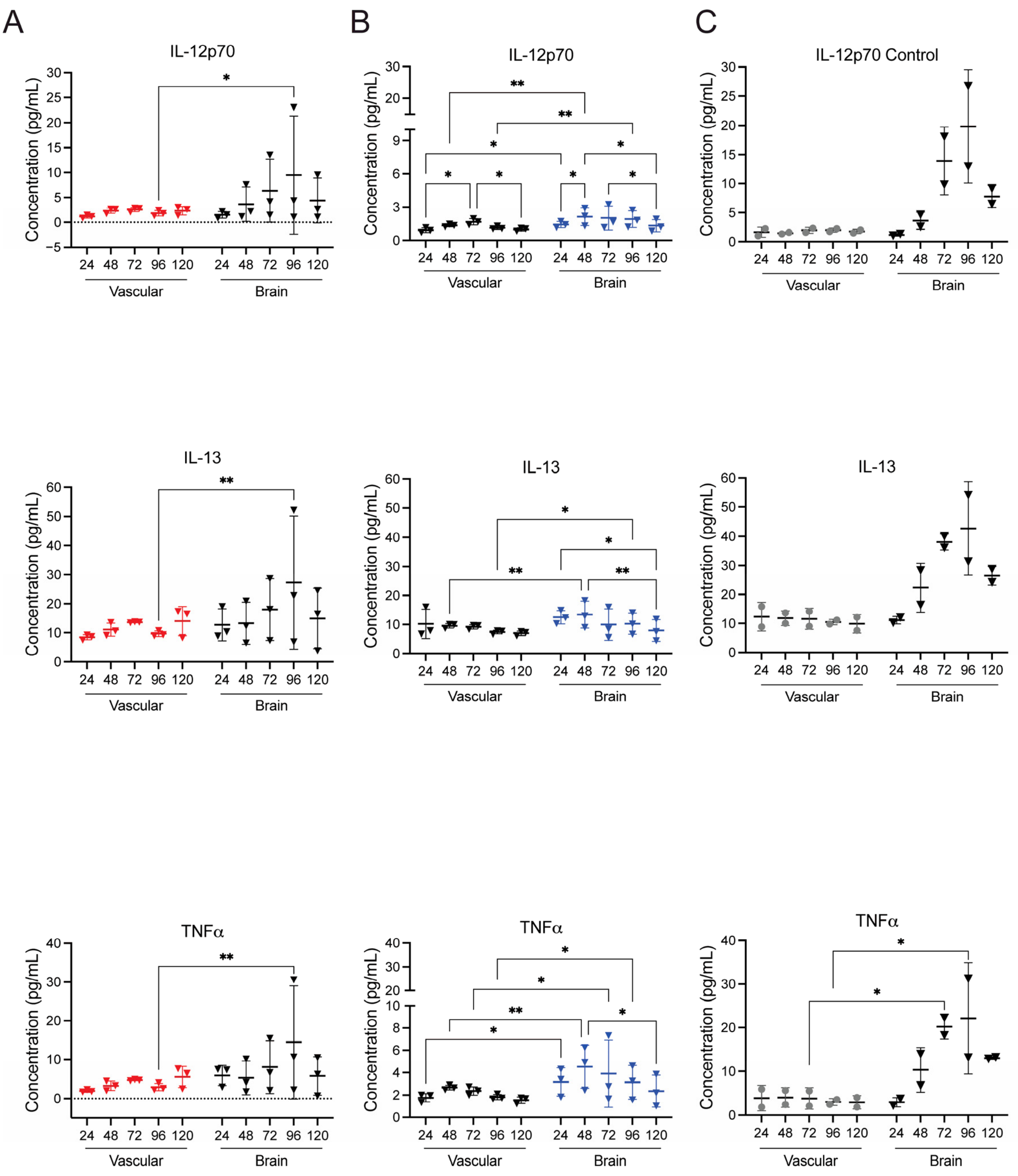

Figure S3. EEEV gNVU VRI and BRI inflammatory cytokine expression. Perfused media collected from vascular and brain outlets gNVUs were analyzed for 10 human inflammatory cytokines (IFN-γ, IL-1β, IL-2, IL-4, IL-6, IL-8, IL-10, IL-12p70, IL-13 and TNF-*α*) using the MSD multiplex assay. (**A**) Data from VRI gNVUs (n = 3) showing all proinflammatory cytokines from the vascular infected side in red and from the brain side represented in black. (**B**) Data from BRI gNVUs (n = 3) showing all proinflammatory cytokines from the vascular side represented in black and from the brain-infected side in blue. (**C**) Data from uninfected control gNVUs (n = 2) cytokines with vascular side represented in light grey and brain side data represented in black. Each sample collected from each timepoint was analyzed as technical duplicates and averaged for each cytokine. All cytokine results were analyzed using MSD Discovery Workbench 4.0 software and represented as pg/mL concentration for each cytokine. Statistical analysis was carried out using a two-way ANOVA test on Prism 10. Significance values are indicated using asterisks for * $p < 0.05$, ** $p < 0.01$, *** $p < 0.001$, and **** $p < 0.0001$, while $p \geq 0.05$ is not significant. The dashed line at $y = 0$ is to show standard deviation (SD) spread.

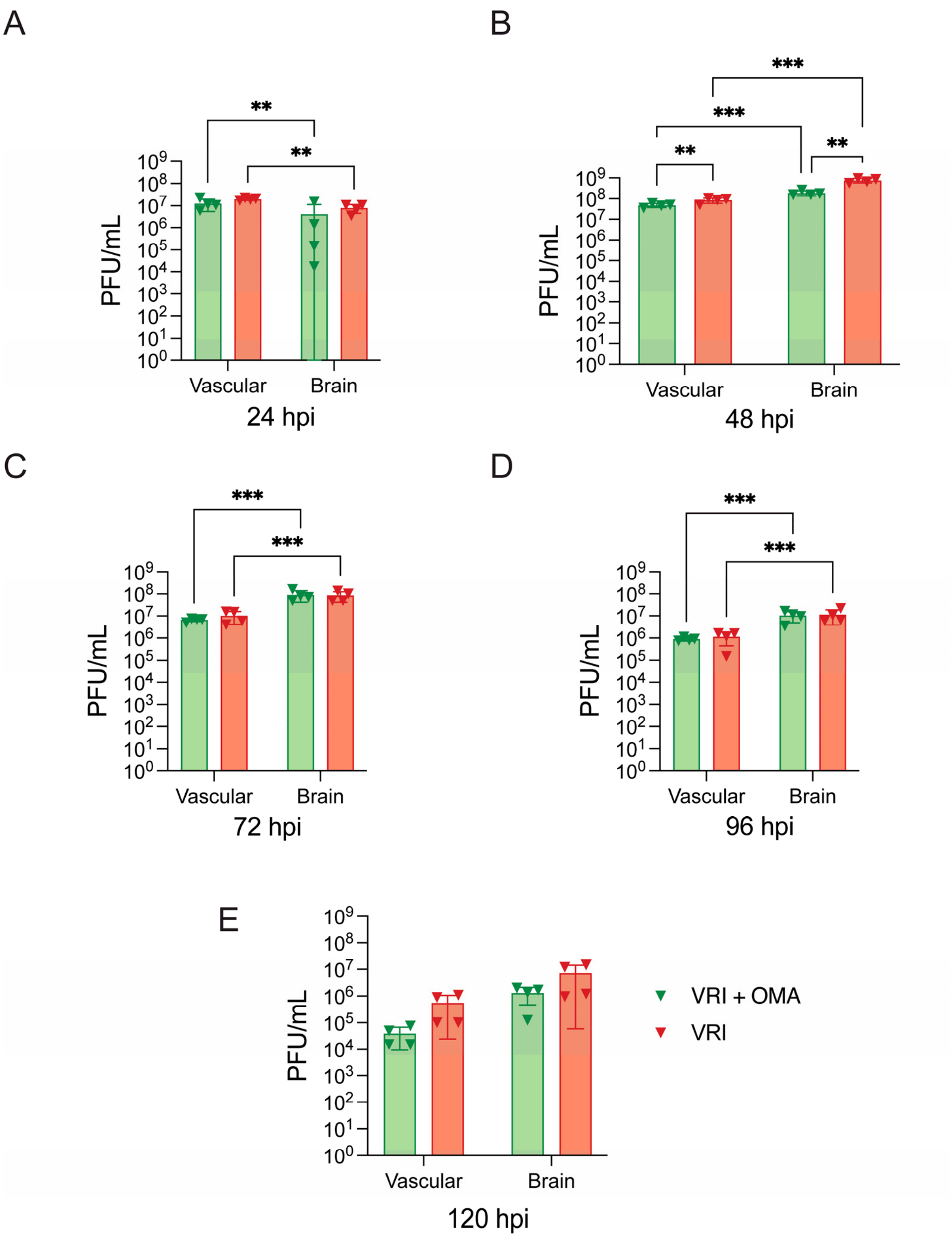

Figure S4. Impact of OMA-treatment on viral load in the gNVU VRI model at five different timepoints. The gNVU chips were either infected with EEEV (MOI 0.1) on the vascular inlet side and left untreated ($n = 4$), or were infected with EEEV (MOI 0.1) on the vascular inlet and treated with OMA (0.5 μM) on the vascular side ($n = 4$). Vascular and brain viral load from both the untreated VRI group (red) and the OMA-treated VRI group (green) were compared and quantified by plaque assay using perfused supernatants collected from vascular outlet chambers at (**A**) 24 hpi, (**B**) 48 hpi, (**C**) 72 hpi, (**D**) 96 hpi and (**E**) 120 hpi. All data represented for each group are from the $n = 4$ gNVU chips. Statistical analysis was carried out using unpaired two-tailed t-tests in GraphPad Prism 10. Significance values are indicated using asterisks for ** $p < 0.01$, and *** $p < 0.001$, while $p \geq 0.05$ is not significant.

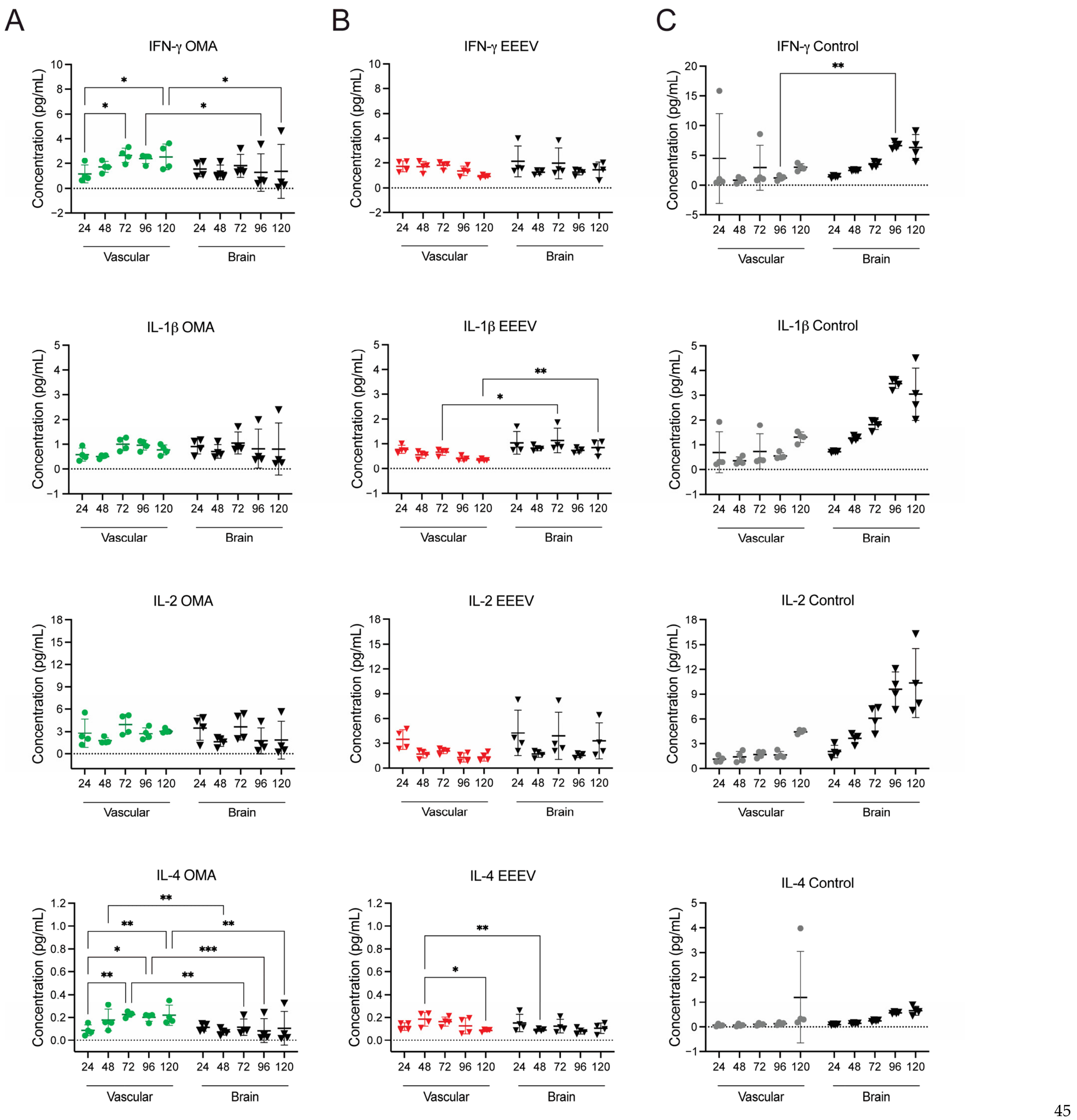
A
B
C
IFN-γ OMA
IFN-γ EEEV
IFN-γ Control
IL-1β OMA
IL-1β EEEV
IL-1β Control
IL-2 OMA
IL-2 EEEV
IL-2 Control
IL-4 OMA
IL-4 EEEV
IL-4 Control
Concentration (pg/mL)
24 48 72 96 120 24 48 72 96 120
Vascular
Brain

45

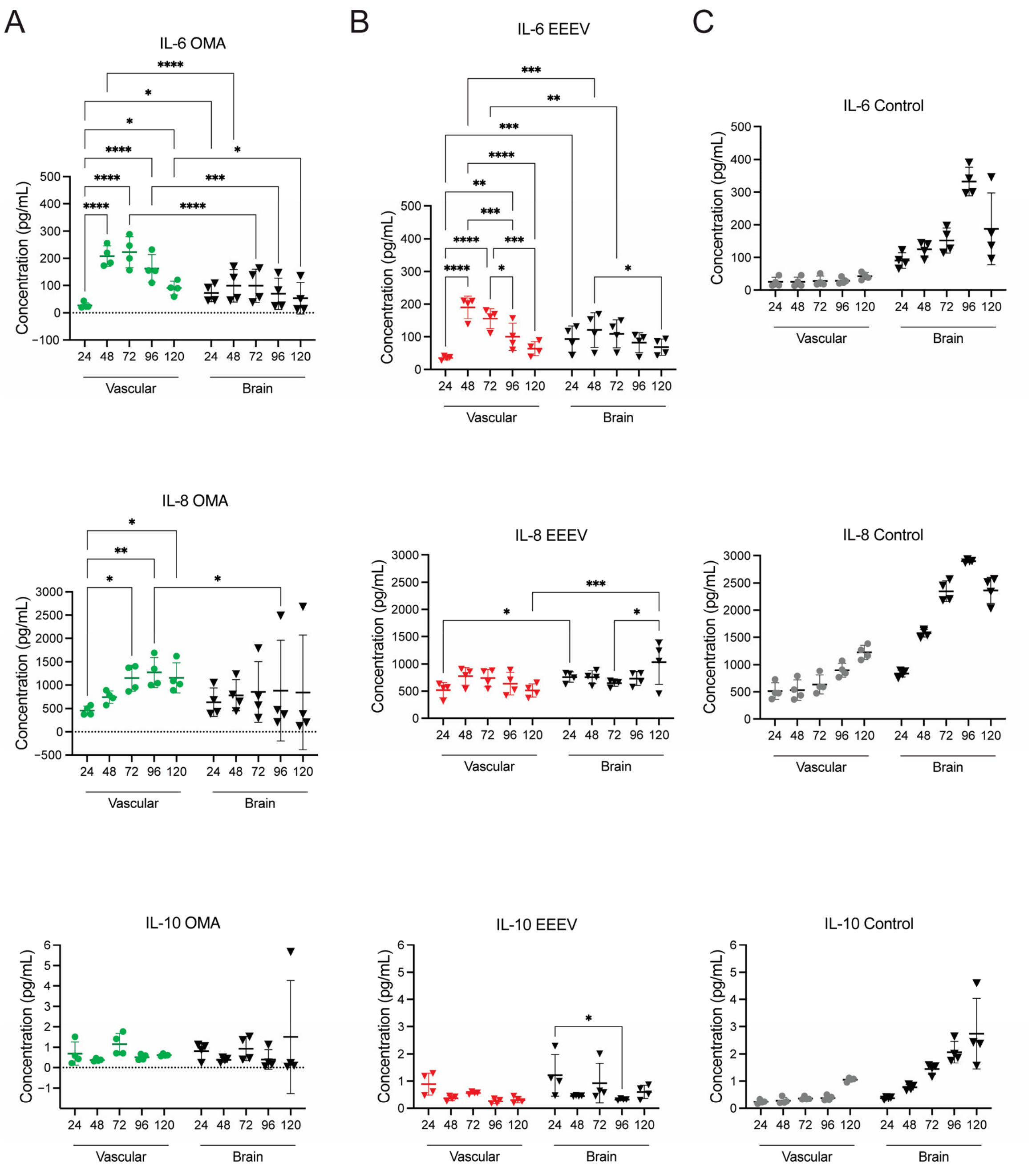
A
B
C
IL-6 OMA
IL-6 EEEV
IL-6 Control
IL-8 OMA
IL-8 EEEV
IL-8 Control
IL-10 OMA
IL-10 EEEV
IL-10 Control
Concentration (pg/mL)
24 48 72 96 120 24 48 72 96 120
Vascular
Brain

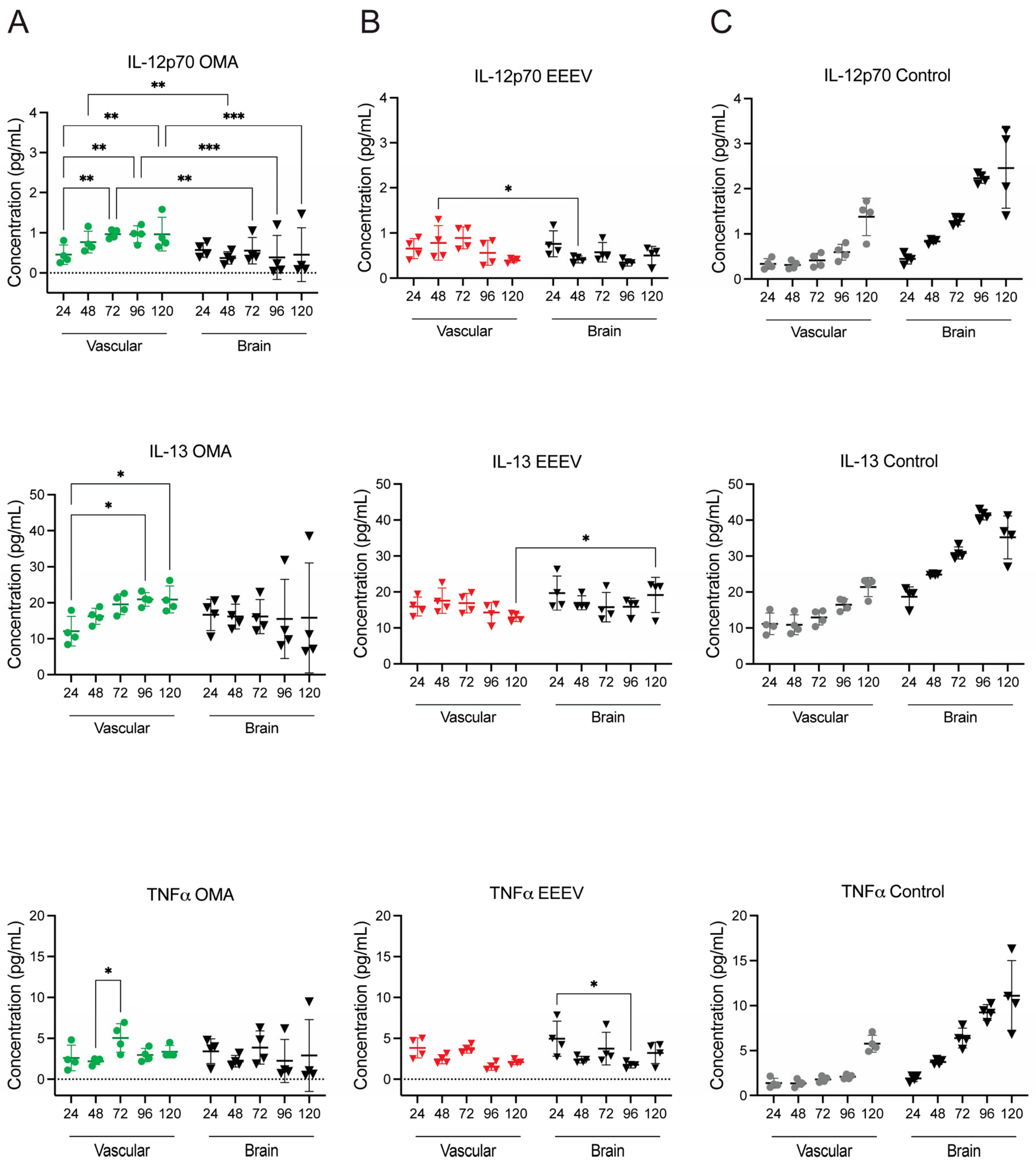

Figure S5. Impact of OMA-treatment on inflammatory cytokine response in EEEV VRI model of gNVU. The gNVU chips were either infected with EEEV (MOI 0.1) on the vascular inlet side and left untreated (n = 4), or were infected with EEEV (MOI 0.1) on the vascular inlet and treated with OMA (0.5 µM) on the vascular side (n = 4). For the control group, gNVU chips were left uninfected and untreated. Perfused media were collected from the vascular and brain outlets of all chips every 24 h until 120 hpi, and were analyzed for 10 inflammatory cytokines (IFN-γ, IL-1β, IL-2, IL-4, IL-6, IL-8, IL-10, IL-12p70, IL-13 and TNF-α) using MSD multiplex assay. (**A**) Data from OMA-treated VRI gNVUs (n = 4) showing all proinflammatory cytokines from the vascular infected side in green and from the brain side represented in black. (**B**) Data from untreated VRI gNVUs (n = 4) showing all proinflammatory cytokines from the vascular infected side in red and from the brain side represented in black. (**C**) Data from uninfected control gNVUs (n = 4) cytokines with the vascular side represented in light grey and the brain side data represented in black. Each sample collected from each timepoint was analyzed as technical duplicates and averaged for each cytokine. All the cytokine results were analyzed using MSD Discovery Workbench 4.0 software and represented as

pg/mL concentration for each cytokine. Statistical analysis was carried out using a two-way ANOVA test on Prism 10. Significance values are indicated using asterisks for * $p < 0.05$, ** $p < 0.01$, *** $p < 0.001$, and **** $p < 0.0001$, while $p \geq 0.05$ is not significant. The dashed line at $y = 0$ is to show standard deviation (SD) spread. 62 63 64 65

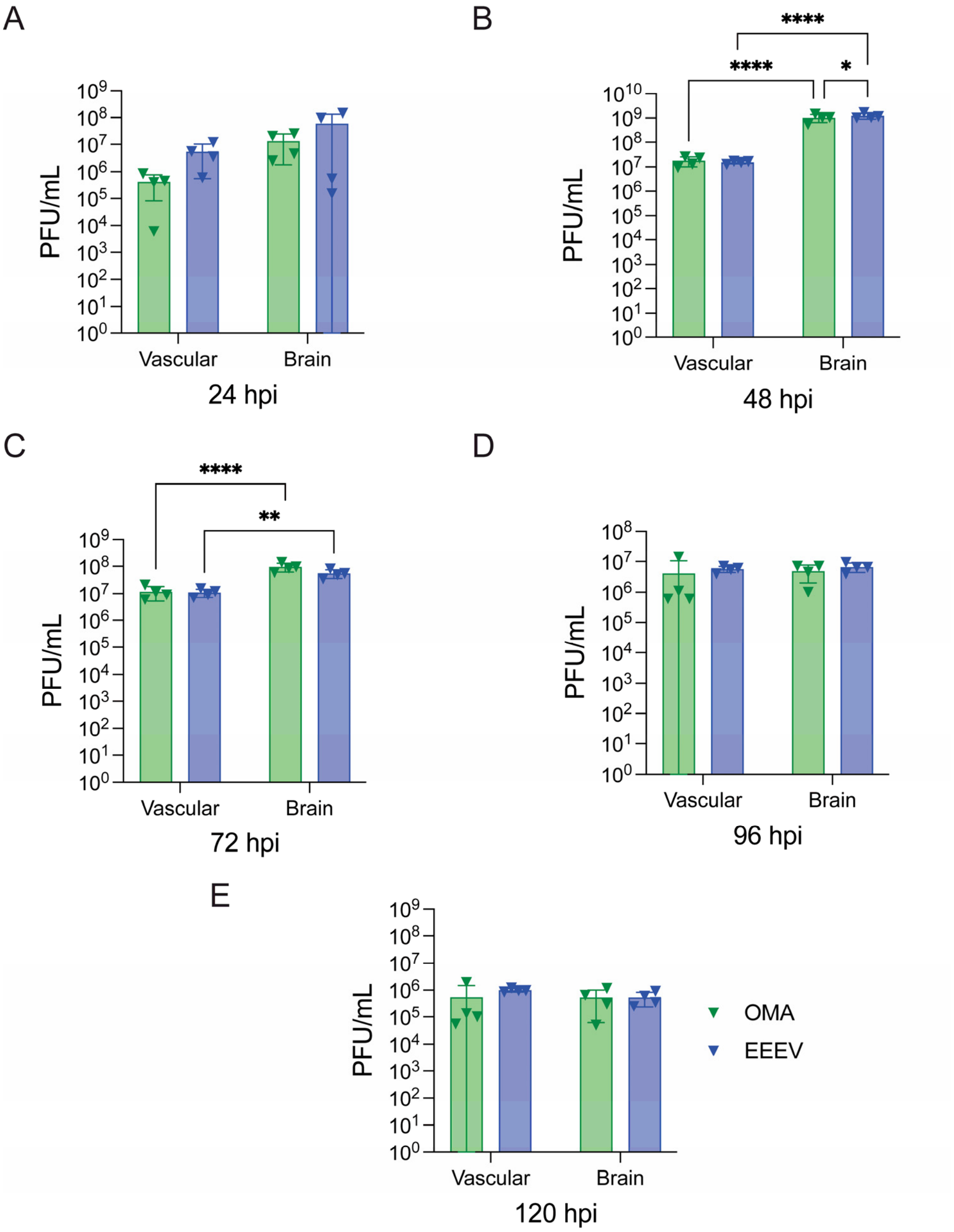

66

Figure S6. Impact of OMA treatment on the viral load in the gNVU BRI model at five different timepoints. The gNVU chips were either infected with EEEV (MOI 0.1) on the brain inlet side and left untreated ($n = 4$), or were infected with EEEV (MOI 0.1) on the brain inlet and treated with OMA (0.5 µM) on the vascular side ($n = 4$). Vascular and brain viral load from untreated BRI group (blue) and OMA-treated BRI group (green) compared and quantified by plaque assay using perfused supernatants collected from vascular outlet chambers at (A) 24 hpi, (B) 48 hpi, (C) 72 hpi, (D) 96 hpi and (E) 120 hpi. All data represented for each group are from $n = 4$ gNVU chips. Statistical analysis was carried out using unpaired two-tailed t-tests in GraphPad Prism 10. Significance values are indicated using asterisks for * $p < 0.05$, ** $p < 0.01$, and **** $p < 0.0001$, while $p \geq 0.05$ is not significant.. 67 68 69 70 71 72 73 74 75

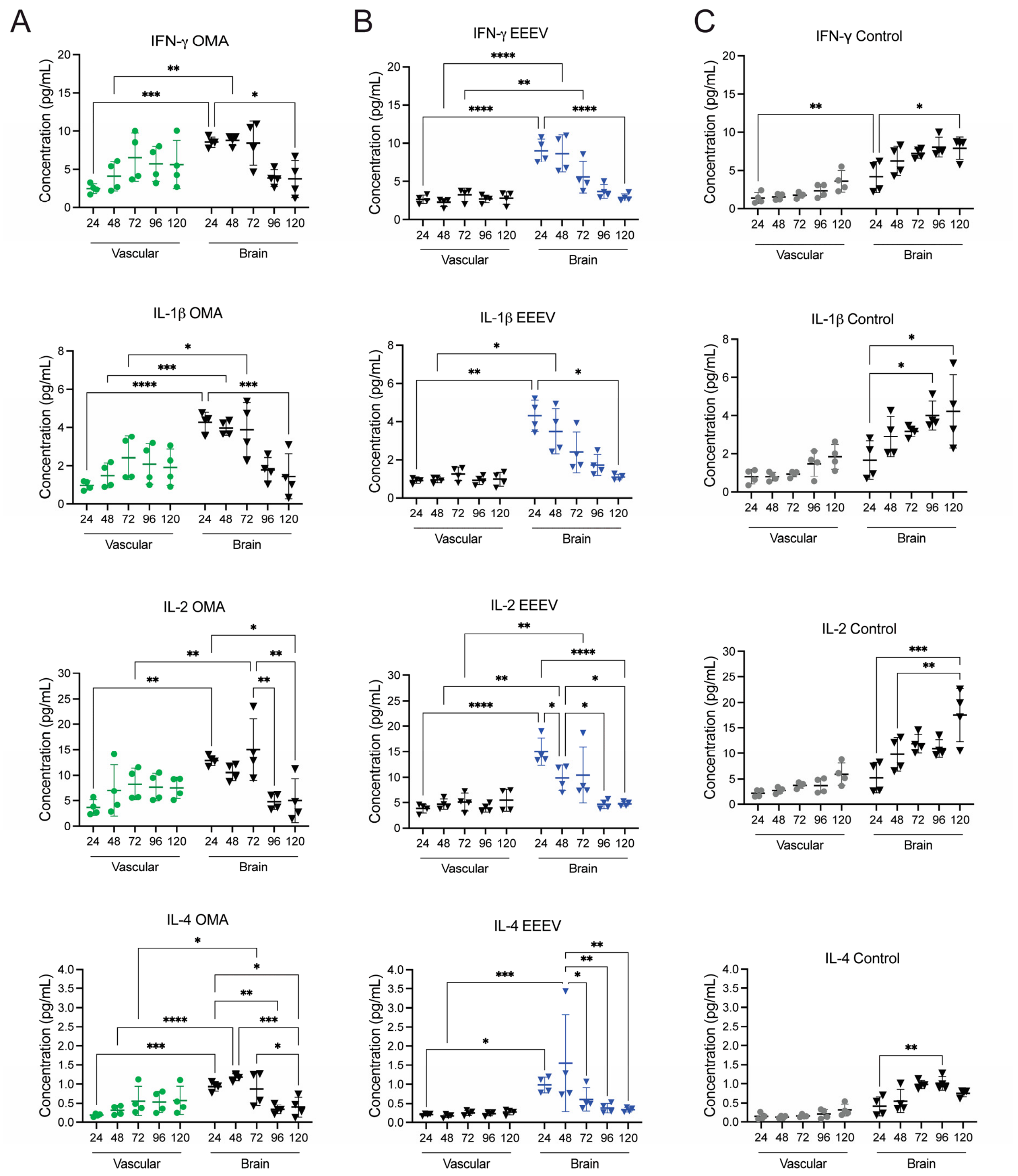
A
B
C
IFN-γ OMA
IFN-γ EEEV
IFN-γ Control
IL-1β OMA
IL-1β EEEV
IL-1β Control
IL-2 OMA
IL-2 EEEV
IL-2 Control
IL-4 OMA
IL-4 EEEV
IL-4 Control
Concentration (pg/mL)
24 48 72 96 120 24 48 72 96 120
Vascular
Brain

76

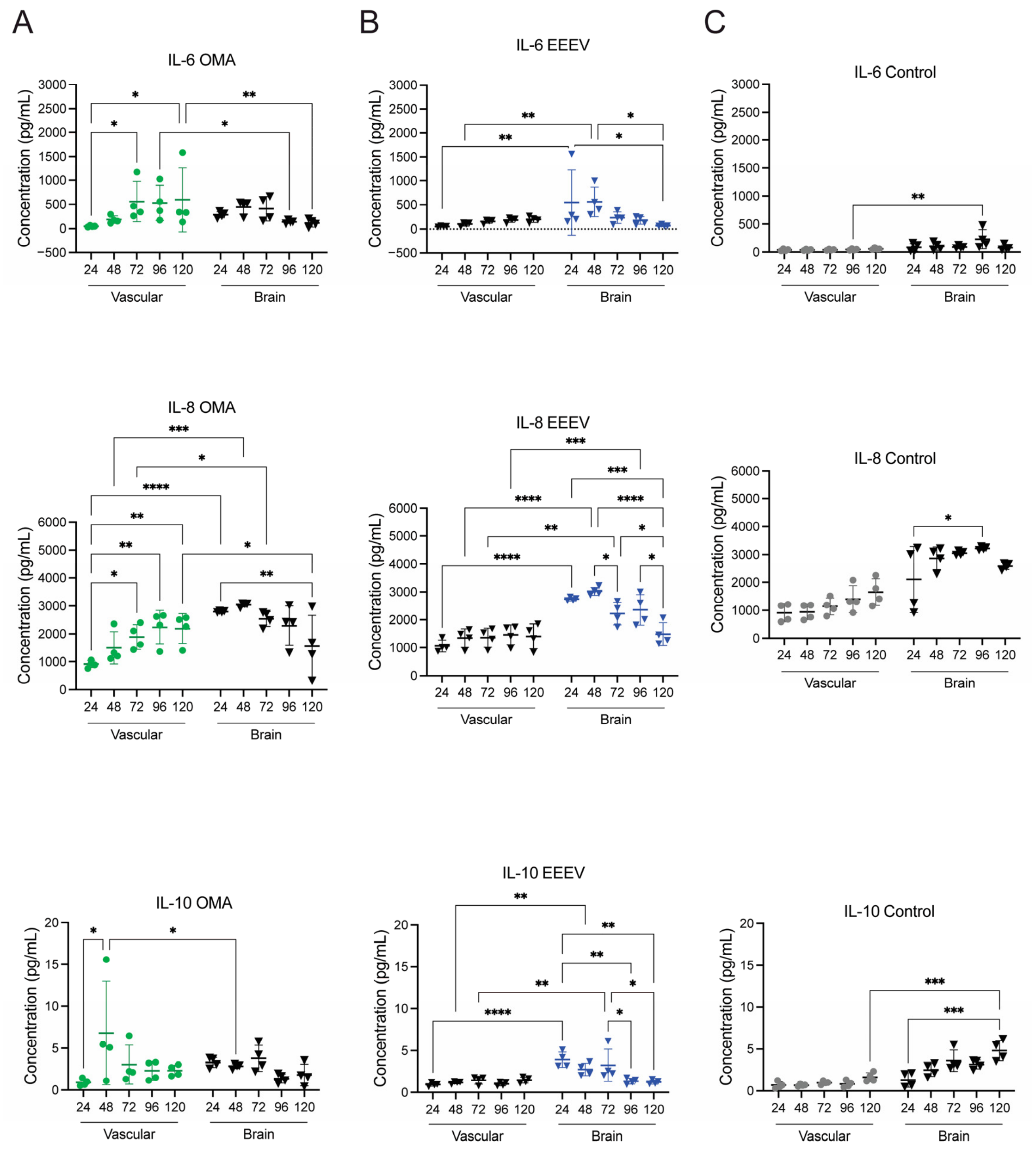
A
B
C
IL-6 OMA
IL-6 EEEV
IL-6 Control
IL-8 OMA
IL-8 EEEV
IL-8 Control
IL-10 OMA
IL-10 EEEV
IL-10 Control
Concentration (pg/mL)
Vascular
Brain
24 48 72 96 120

77

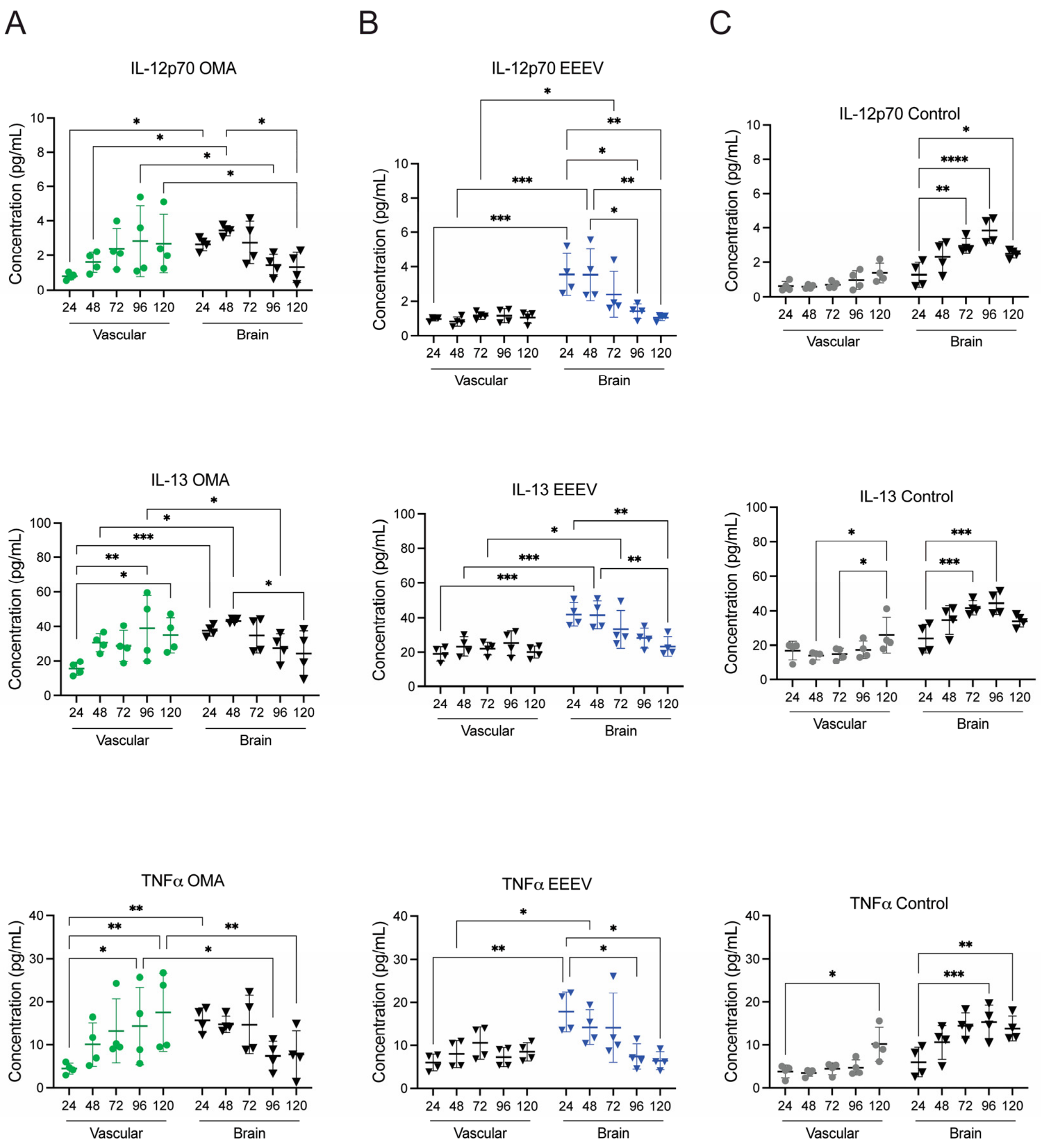

Figure S7. Impact of OMA treatment on the inflammatory cytokine response in the EEEV BRI model of gNVU. The gNVU chips were either infected with EEEV (MOI 0.1) on the brain inlet side and left untreated (n = 4), or were infected with EEEV (MOI 0.1) on the brain inlet and treated with OMA (0.5 μM) on the vascular side (n = 4). For the control group, gNVU chips were left uninfected and untreated. Perfused media were collected from the vascular and brain outlets of all chips every 24 h until 120 hpi, and were analyzed for 10 inflammatory cytokines (IFN-γ, IL-1β, IL-2, IL-4, IL-6, IL-8, IL-10, IL-12p70, IL-13 and TNF-α) using MSD multiplex assay. (**A**) Data from OMA-treated BRI gNVUs (n = 4) showing all inflammatory cytokines from the vascular infected side in green and from the brain side represented in black. (**B**) Data from untreated BRI gNVUs (n = 4) showing all proinflammatory cytokines from the vascular infected side in black and from the brain side represented in blue. (**C**) Data from uninfected control gNVUs (n = 4) cytokines with vascular side represented in light grey and brain side data represented in black. Each sample collected from each timepoint was analyzed as technical duplicates and averaged for each cytokine. All cytokine results were analyzed using MSD Discovery Workbench 4.0 software and represented as pg/mL concentration for each cytokine. Statistical analysis was carried out using a two-way ANOVA test on Prism 10. Significance values are indicated using asterisks for * $p < 0.05$, ** $p < 0.01$, *** $p < 0.001$, and **** $p < 0.0001$, while $p \geq 0.05$ is not significant. The dashed line at $y = 0$ is to show standard deviation (SD) spread.
